# ROCker Models for Reliable Detection and Typing of Short-Read Sequences Carrying β-Lactamase Genes

**DOI:** 10.1128/msystems.01281-21

**Published:** 2022-05-31

**Authors:** Si-Yu Zhang, Brittany Suttner, Luis M. Rodriguez-R, Luis H. Orellana, Roth E. Conrad, Fang Liu, Jessica L. Rowell, Hattie E. Webb, Amanda J. Williams-Newkirk, Andrew Huang, Konstantinos T. Konstantinidis

**Affiliations:** a School of Civil and Environmental Engineering, Georgia Institute of Technology, Atlanta, Georgia, USA; b School of Ecological and Environmental Sciences, East China Normal University, Shanghai, China; c School of Biological Sciences, Georgia Institute of Technology, Atlanta, Georgia, USA; d Department of Microbiology and Digital Science Center, University of Innsbruck, Innsbruck, Tyrol, Austria; e Max-Planck-Institut für Marine Mikrobiologie, Bremen, Germany; f Partnership for an Advanced Computing Environment, Georgia Institute of Technology, Atlanta, Georgia, USA; g Enteric Diseases Laboratory Branch, Centers for Disease Control and Prevention, Atlanta, Georgia, USA; h Weems Design Studio, Suwanee, Georgia, USA; Marquette University

**Keywords:** β-lactamases, short reads, ROCker models, high F1 score

## Abstract

Identification of genes encoding β-lactamases (BLs) from short-read sequences remains challenging due to the high frequency of shared amino acid functional domains and motifs in proteins encoded by BL genes and related non-BL gene sequences. Divergent BL homologs can be frequently missed during similarity searches, which has important practical consequences for monitoring antibiotic resistance. To address this limitation, we built ROCker models that targeted broad classes (e.g., class A, B, C, and D) and individual families (e.g., TEM) of BLs and challenged them with mock 150-bp- and 250-bp-read data sets of known composition. ROCker identifies most-discriminant bit score thresholds in sliding windows along the sequence of the target protein sequence and hence can account for nondiscriminative domains shared by unrelated proteins. BL ROCker models showed a 0% false-positive rate (FPR), a 0% to 4% false-negative rate (FNR), and an up-to-50-fold-higher F1 score [2 × precision × recall/(precision + recall)] compared to alternative methods, such as similarity searches using BLASTx with various e-value thresholds and BL hidden Markov models, or tools like DeepARG, ShortBRED, and AMRFinder. The ROCker models and the underlying protein sequence reference data sets and phylogenetic trees for read placement are freely available through http://enve-omics.ce.gatech.edu/data/rocker-bla. Application of these BL ROCker models to metagenomics, metatranscriptomics, and high-throughput PCR gene amplicon data should facilitate the reliable detection and quantification of BL variants encoded by environmental or clinical isolates and microbiomes and more accurate assessment of the associated public health risk, compared to the current practice.

**IMPORTANCE** Resistance genes encoding β-lactamases (BLs) confer resistance to the widely prescribed antibiotic class β-lactams. Therefore, it is important to assess the prevalence of BL genes in clinical or environmental samples for monitoring the spreading of these genes into pathogens and estimating public health risk. However, detecting BLs in short-read sequence data is technically challenging. Our ROCker model-based bioinformatics approach showcases the reliable detection and typing of BLs in complex data sets and thus contributes toward solving an important problem in antibiotic resistance surveillance. The ROCker models developed substantially expand the toolbox for monitoring antibiotic resistance in clinical or environmental settings.

## INTRODUCTION

Genes encoding β-lactamases (BLs) confer resistance to a widely prescribed class of antibiotics, the β-lactams (1). These β-lactam-degrading enzymes represent a major public health threat due to their ability to inactivate clinically important β-lactams and rapid dissemination of β-lactamase genes among pathogenic microorganisms (2, 3). Mobilization of BL genes from environmental organisms into pathogens also occurs (4), since β-lactams are naturally produced compounds (5–7), and is also relevant for public health. Currently, more than 1,800 BL variants have been described (8). BL variants are classified into four classes based on conserved active-site amino acid motifs (Ambler classification) (9) or multiple classes based on functional characteristics and substrate/inhibitor profiles (Bush-Jacoby-Medeiros classification) (10). According to the Ambler classification system, BL variants are classified into four molecular classes: A, B, C, and D (9). Among the Ambler classes, classes A, C and D are serine BLs, which hydrolyze β-lactams by forming an acyl enzyme intermediate through an active-site serine, whereas class B enzymes are metallo-β-lactamases (MBLs) and utilize at least one active-site zinc ion to hydrolyze their substrates (11).

Assessing presence of β-lactamases and their specific variants with next-generation sequencing (NGS) technologies in isolate genomes and metagenomes could help guide treatment selection and enable improved surveillance of BLs in both clinical and environmental settings (12–14). However, identifying short reads carrying BL genes remains challenging due to the frequent sharing of amino acid functional domains and motifs between proteins encoded by target antimicrobial resistance genes (ARGs) like BL genes and non-BL or other (non-ARG) gene sequences (2, 3). Full-length gene sequences assembled as part of genomes or metagenomes generally represent less of a problem in this respect, although the high sequence divergence often observed between and within BL classes represents another major challenge for identification of short-read or full-length gene sequences carrying BLs (8, 12). For instance, distinct BLs, even of the same class, frequently share less than 40% amino acid identity across their sequences, which is problematic for detecting homologous sequences (13).

Identification of ARG-carrying reads typically relies on sequence similarity searches against curated databases, such as ResFinder (14), the Comprehensive Antibiotic Resistance Database (CARD) (15), the Antibiotic Resistance Gene Database (ARDB) (16), and UniProt (Universal Protein Resource) databases, or the use of ARGs-OAP and DeepARG tools (12, 17, 18). Due to the continuous release of genomes and metagenomes carrying novel BL variants (5), these curated databases are not updated in a timely fashion. Further, BL sequences are frequently annotated inconsistently or erroneously in the public domains (19), meaning that sequences remain unassigned to a family or class, or sequences that are related but do not encode BLs, such as lactam-binding (but not hydrolyzing) sequences, are identified as BLs. For these reasons, as well as the technical challenges mentioned above related to shared amino acid domains and motifs, the tools and databases available are far from ideal for detecting BL-encoding genes from metagenomic samples, especially for sequences carried on short reads (12, 17, 18). Hence, a reliable approach to detecting BLs, including novel and divergent variants, in short-read (unassembled) metagenomic data sets is needed. It is important to note that these issues are also relevant for amplicon sequencing data sets, such as those that result from PCR assays with broad-specificity BL primers. In particular, it remains challenging to distinguish between BL sequences (specific priming) and non-BL sequences produced in such amplicon data sets due to nonspecific priming (20, 21). Regions of high sequence identity also often make it difficult to distinguish accurately between multiple BLs in both amplicon and shotgun short-read metagenome data (22, 23).

To address the technical limitations in detecting BLs in short-read metagenomic shotgun or amplicon sequences, we built ROCker models for BLs and evaluated them with mock 150-bp- and 250-bp-read data sets of known composition. Instead of relying on fixed e-value thresholds for the whole alignment, as is typically the case in similarity searches for detecting ARG-carrying sequences, which can return an unknown number of false positives and negatives, ROCker identifies position-specific, most-discriminant bit score thresholds in sliding windows along the sequence of the target protein sequence (e.g., BL) using the receiver operating characteristic (ROC) curve. Hence, ROCker can account for nondiscriminative domains shared by unrelated proteins (24). We previously reported that ROCker often shows more than a 50-fold-lower false discovery rate (FDR) than the common practice of using fixed e-values or hidden Markov model (HMM)-based searches for genes of the microbial nitrogen cycle (24). Here, we show that our ROCker models can reliably detect and type BLs in short-read metagenomes with similar differences in FDR and a high accuracy (F1 score) compared to alternative approaches (F1 = ~1 versus 0.02 to 0.86). Specifically, we built ROCker models for all BL classes, i.e., classes A, B, C, and D of the Ambler classification system, as an example of the between-class resolution that ROCker can provide. We also built ROCker models for TEM (Temoniera β-lactamases, in class A), as an example of the within-class resolution of a highly conserved family of BLs that ROCker models can provide. To avoid repetition of our methodology and results, we present the results for two representative ROCker models, class D BLs (oxacillinase β-lactamases [OXA]) and TEM, in the article; we provide the remaining models in the supplemental material.

## RESULTS

We describe below the results of each step required to build and evaluate ROCker models for TEM-like variants and class D BL-like variants as representative examples of phylogenetically narrow and diverse protein families, respectively. Illumina-like simulated short-read data sets of known composition (mock data sets) were used in the evaluation.

### Assessment of different options for building ROCker models based on training data sets.

**FIG 1:**
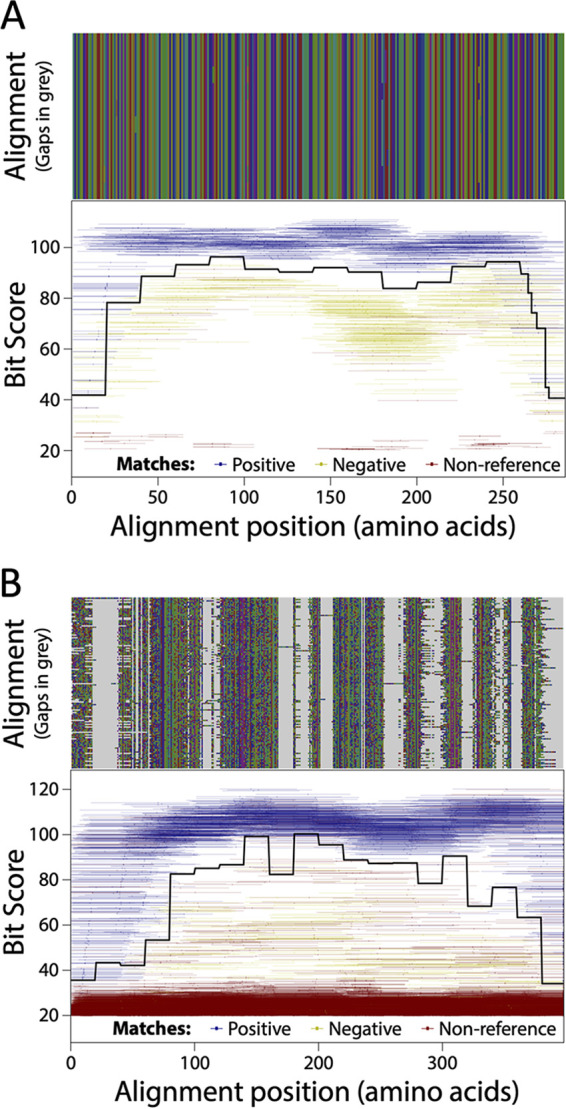
Plots of the 150-bp ROCker model of (A) TEM using a DIAMOND search and (B) the class D BL after trimming of the 5′ conserved end of the alignment. (Top) Sequence alignments with amino acids in different colors and gaps in light gray. (Bottom) Bit scores (*y* axis) of the reads of the 150-bp training data set against the positive-reference sequences used to build the ROCker model carrying positive references (blue), negative references (yellow), or nontarget sequences (red). Each matching read is represented as a line based on the coordinates of the alignment of the positive-reference sequences to which the read maps, and dots represent the midpoint of each read. The solid black traversing line represents the calculated ROCker best bit score thresholds for consecutive windows of variable length. The sensitivity, specificity, and accuracy were 98.8%, 99.2%, and 99.0% for the TEM model and 99.1%, 99.8%, and 99.8% for the class D BL model.

The best-performing ROCker models based on model training data sets constructed from positive and negative (when available) sequences were observed when using DIAMOND (detailed in “Technical challenges and tips when using a ROCker model” below). We used the following terms and definitions to determine the quality of results: true positive (TP); false positive (FP); false negative (FN); positives (P) = TP + FP; negatives (N) = TN + FN; false negative rate (FNR) = FN/(TP + FN); false positive rate (FPR) = FP/P; sensitivity = 1 − FNR = TP/(TP + FN); specificity = 1 − FPR = TP/P; and accuracy = (TP + TN)/(P + N). Sensitivity, specificity, and accuracy were 98.8%, 99.2%, and 99.0%, respectively, for the 150-bp TEM ROCker model using DIAMOND (Fig. 1A). For the 250-bp TEM ROCker model, the same statistics were 96.5%, 99.3%, and 97.9%, respectively (Fig. S2A). For the ROCker model of class D BLs, the best performance was achieved after removing (trimming) the first 100 residues of the protein alignment, which were not specific enough for class D BLs (i.e., the bit score was similar between the target and nontarget protein sequences). The sensitivity, specificity, and accuracy of the trimmed ROCker models were 99.1%, 99.8%, and 99.8%, respectively, for the 150-bp model (Fig. 1B) and 97.4%, 99.9%, and 99.9%, respectively, for the 250-bp model (Fig. S2B). Hence, these models were used in the subsequent analyses.

10.1128/mSystems.01281-21.4 FIG S2 (A and B) Plots of the 250-bp ROCker model of (A) TEM using a DIAMOND search and (B) the class D BL model after trimming of the 5′ conserved end of the alignment. The sensitivity, specificity, and accuracy were 96.5%, 99.3%, and 97.9% for the TEM and 97.4%, 99.9%, and 99.9% for the class D BL models, respectively. (C) Plot of the 150-bp ROCker model of TEM using a BLASTx search with default settings. According to the plot, there is an unexpected drop of the bit score threshold around positions 150 to 250 of the alignment. Further examination showed that the corresponding target reads were assigned a lower bit score than expected due to a BLAST filter for low-complexity sequences. Download FIG S2, PDF file, 0.6 MB. Copyright © 2022 Zhang et al. 2022 Zhang et al. https://creativecommons.org/licenses/by/4.0/ This content is distributed under the terms of the Creative Commons Attribution 4.0 International license.

### Building mock data sets and metrics for evaluating performance.

**FIG 2:**
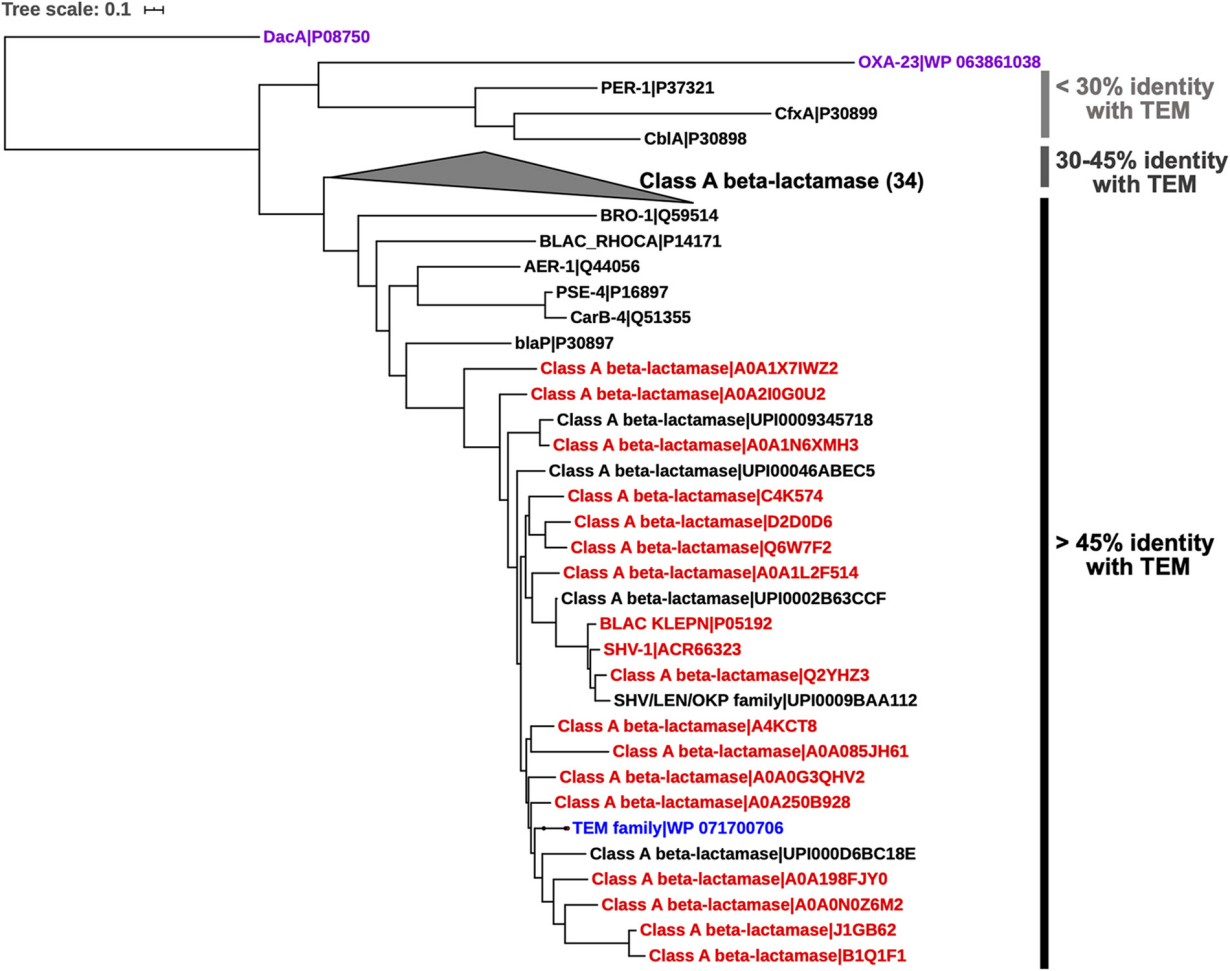
Phylogenetic relationships of TEM (in blue) with other families of the class A β-lactamases. d-alanyl-d-alanine carboxypeptidase (DacA; outgroup) and OXA-23 (class D β-lactamase) were included for comparison (in purple, at the top). The references were first clustered by 90% amino acid identity using cd-hit, and only one representative per resulting cluster was included. The phylogenetic tree was constructed using maximum likelihood in RAxML version 7.7.2 with the GTRGAMMA model. The references in red (β-lactamases that are not of the TEM family) were included as negative references used in building the ROCker model. See Materials and Methods for details on reference sequence selection.

Mock metagenomic-like data sets were constructed to evaluate the built ROCker models based on training data sets and compare results against other tools. In short, the mock data set typically included a couple hundred reads (nucleotide sequences) originating from one or two target BL sequences within a background of medium to high genome complexity (see Materials and Methods for details), including about a dozen related but nontarget sequences; the target BL sequences differed between models (e.g., TEM versus class D), but the remainder of the data sets were similar. The tools were evaluated for their ability to detect (identify) the target reads and not identify as target the nontarget reads (see also below). Specifically, the 150-bp mock data set contained 107 *bla*_TEM_ and 331 class D BL gene-carrying reads that were generated *in silico* from one TEM and two class D BL reference nucleotide sequences using Grinder (25), whereas the 250-bp mock set contained 98 *bla*_TEM_ and 316 class D BL gene-carrying reads. The two class D BLs shared 20% amino acid identity with each other, and both have a homolog with ~90% amino acid identity but were not themselves present in the reference class D BL tree or in the positive-reference sequences used to build the ROCker and hidden Markov models. Only one TEM was included in the mock data set, because there was not substantial diversity among TEM protein sequences in UniProt. The non-target-related sequences used in the mock data set included one class B BL, two class C BLs that share 47% amino acid identity to each other, a d-alanyl-d-alanine carboxypeptidase (DacA), and a hydroxyacylglutathione hydrolase (GloB) that shared some of the conserved domains with target BLs and showed 10% to 50% sequence amino acid identity to different classes of BLs, in addition to reads derived from the other genes in the genomes that encoded these nontarget sequences. In order to increase the complexity of the TEM mock data set, another 10 non-target-related references were included that represent different clades of the phylogenetic tree of class A BL variants with one or two representatives each (Fig. 2). These nontarget TEM sequences shared domains and motifs with TEM families at 30% to 80% sequence amino acid identity and were also used as class A BL target sequences for the class A BL data sets. UniProt identifiers for BL nucleotide sequences and their host genome sequences used to create the mock data sets are listed in Data Set S1, sheet 3. Mock data sets are also available on our website that hosts the ROCker models.

10.1128/mSystems.01281-21.2 DATA SET S1 (Sheet 1) Information on positive- and negative-reference sets for TEM ROCker building. (Sheet 2) Information on positive- and negative-reference sets for class D β-lactamase ROCker building. (Sheet 3) Information on β-lactamase protein sequences and their related genomes for MOCK data set building. (Sheet 4) Performance of the TEM ROCker model compared with the similarity search with a fix e-value of 1E-2, 1E-5, 1E-10, 1E-20 and 1E-30, and HMMs (e-value < 0.1) downloaded from FunGene and Pfam databases or customer build using the same positive-reference sets taken by ROCker. (Sheet 5) Performance of the class D β-lactamase ROCker model compared with the similarity search with a fixed e-value of 1E−2, 1E−5, 1E−10, 1E−20, and 1E−30 and HMMs (e-value < 0.1) downloaded from FunGene and Pfam databases or custom built using the same positive-reference sets taken by ROCker. Download Data Set S1, XLSX file, 0.03 MB. Copyright © 2022 Zhang et al. 2022 Zhang et al. https://creativecommons.org/licenses/by/4.0/ This content is distributed under the terms of the Creative Commons Attribution 4.0 International license.

**FIG 3:**
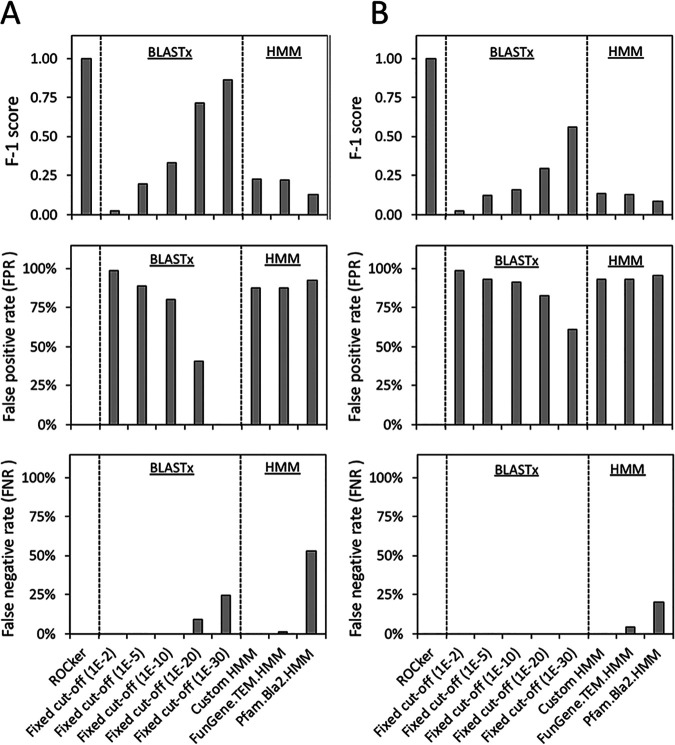
Performance of the TEM ROCker model on 150-bp (A) and 250-bp (B) mock data sets compared to alternative methods. Alternative methods included BLASTx searches using a fixed E value (10^−2^, 10^−5^, 10^−10^, 10^−20^, and 10^−30^) and HMM-based searches using HMMs (E value < 0.1) downloaded from FunGene or Pfam databases or a custom-built HMM using the positive-reference sequence set used to build the ROCker model (*x* axis labels). F1 score [2 × precision × recall/(precision + recall)] (top), FPR [FP/(TP + FP)] (middle), and FNR [FN/(TP + FN)] (bottom) are shown.

**FIG 4:**
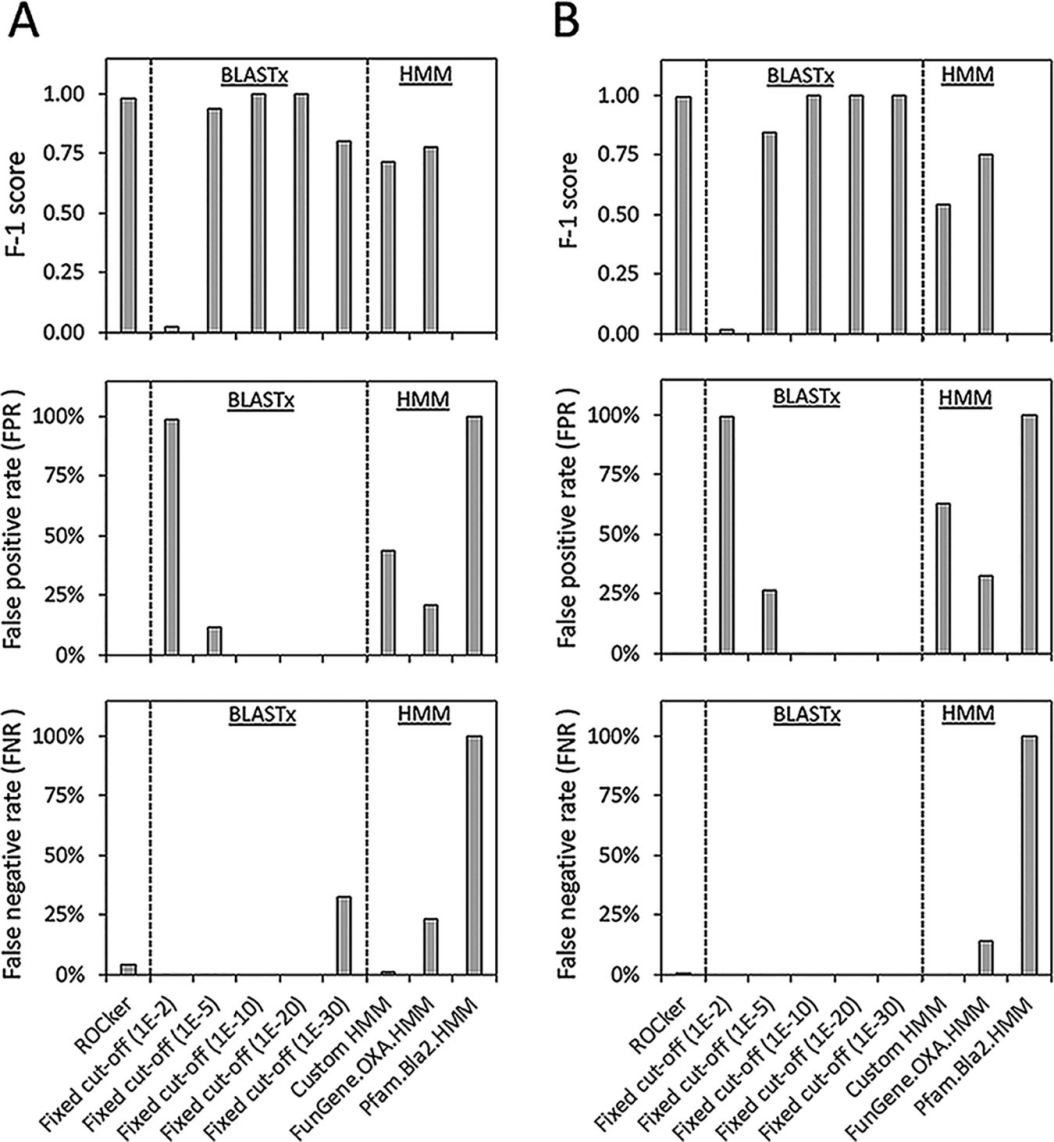
Performance of the class D BL ROCker model on 150-bp (A) and 250-bp (B) mock data sets compared to alternative methods. The methods and parameters were as described for Fig. 3, and the same statistics are shown.

The performance of ROCker models with 150-bp and 250-bp simulated data sets were evaluated and compared with alternative approaches employing a fixed e-value: either a BLASTx search with e-values of <10^−2^, 10^−5^, 10^−10^, 10^−20^, and 10^−30^ or a search via hmmsearch using BL HMMs with an e-value of <0.1. Only reads with >90% of their length carrying the target BL genes were considered target reads (positive matches); reads carrying a shorter fragment of the target gene sequence were not considered targets or nontargets. For each method evaluated, each target read was either correctly identified as a target BL of interest (true positive [TP]) or incorrectly identified as a nontarget sequence (false negative [FN]). Nontarget reads were those that carried any sequence other than the target BL gene, i.e., a gene sequence for an unrelated or related but functionally distinct protein. For each method evaluated, these reads were classified as false-positive (FP) matches if they were incorrectly identified as carrying the target BL; otherwise, the reads were considered true negatives (TN). Prediction accuracy of each method was measured as the false-negative rate (FNR) [FN/(TP + FN)], which identifies the failures to detect target sequences; the false-positive rate (FPR) [FP/(TP + FP)], which identifies the failures to exclude non-target sequences; precision [TP/(TP + FP)], which represents how reliable the detected reads are; recall [TP/(TP + FN)], which represents how efficient the detection of all target reads is; and the F1 score [2 × precision × recall/(precision + recall)], a summary metric that combines precision and recall. Note that the F1 score is more comprehensive but cannot distinguish different failures in the results, and therefore, we report all other metrics. ROCker was more accurate (higher F1 score), in general, in detecting reads carrying target BLs from the simulated mock data sets (150 bp and 250 bp) than alternative approaches (Fig. 3 and Data Set S1, sheet 4, for TEM; Fig. 4 and Data Set S1, sheet 5, for class D BLs).

### Comparison of ROCker models to alternative approaches. (i) TEM ROCker model.

For detection of *bla*_TEM_-carrying reads, ROCker had perfect precision (1.00), recall (1.00), and F1 score (1.00) for both the 150-bp and 250-bp mock data sets, i.e., no FP and FN reads were called. BLASTx searches had relatively low F1 scores, ranging from 0.02 to 0.86 depending on the e-value threshold applied, and detected many more FP and FN reads (0 to 9,681 and 0 to 26, respectively, at different e-values) compared to ROCker (Fig. 3). With decreasing e-values from 10^−2^ to 10^−30^ in the BLASTx search (higher stringency), the FPR decreased from 99% to 0% based for the 150-bp-read-length data set, but a concomitant increase of FNR from 0% to 24% was also observed. For the 250-bp-read-length data set, no FN was detected by BLASTx searches, but the FPR was always high, e.g., 99% at the 10^−2^ and 61% at the 10^−30^ e-value. The HMMs, either downloaded from the FunGene database or custom built using the same positive references for ROCker, had similar performance. For instance, the custom-built HMM had an F1 score of 0.22 and FPR of 87% for the 150-bp data set, and values of 0.13 and 93%, respectively, for the 250-bp data set. A slightly higher FNR was observed for the TEM HMM downloaded from FunGene compared to the custom-built HMM, i.e., 1% versus 0% and 4% versus 0% for the 150-bp and 250-bp data sets, respectively. The BL HMM downloaded from the Pfam database is not specific for TEM detection (but targets all BLs) and therefore had a lower F1 score than the TEM-specific HMM, i.e., 0.13 versus 0.22 and 0.08 versus 0.13 for the 150-bp and 250-bp data sets, respectively, with a high FPR (93% versus 87% and 96% versus 93% for the 150-bp and 250-bp data sets, respectively) and FNR (53% versus 0% and 20% versus 0% for the 150-bp and 250-bp data sets, respectively) (Data Set S1, sheet 4).

### (ii) Class D BL ROCker model.

For detection of class D BL gene-carrying reads, ROCker had perfect precision (1.00) and nearly perfect recall (0.96 and 0.99 for the 150-bp and 250-bp data sets, respectively) and F1 scores (0.98 and 1.00 for the 150-bp and 250-bp data sets, respectively). In contrast, the F1 scores ranged from 0.02 to 1.00 for BLASTx searches depending on the e-value threshold applied (F1 score = 1.00 at an e-value of 10^−30^), and 0.54 to 0.78 for different HMMs (Fig. 4). A much higher FPR was observed for HMMs and BLASTx searches at an e-value of 10^−2^ than for ROCker, i.e., 21% to 100% versus 0% for the 150-bp data set and 33% to 100% versus 0% for the 250-bp data sets. The more stringent e-value thresholds at 10^−5^, 10^−10^, 10^−20^ and 10^−30^ for BLASTx searches performed well overall and actually had slightly lower FNR than ROCker, i.e., 0% versus 4% for the 150-bp data set and 0% versus 1% for the 250-bp data set. The HMM downloaded from the FunGene database performed slightly better than the custom-built HMM, with a higher F1 score; i.e., 0.78 versus 0.72 for the 150-bp data set, and 0.75 versus 0.54 for the 250-bp data set (Data Set S1, sheet 5). The BL HMM downloaded from Pfam was unable to detect any class D BL gene-carrying reads, likely because the model was not trained to include class D BLs despite being described as a general BL HMM.

### Phylogenetic placement of identified reads to validate results and assess false positives. (i) *bla*_TEM_-carrying reads.

**FIG 5:**
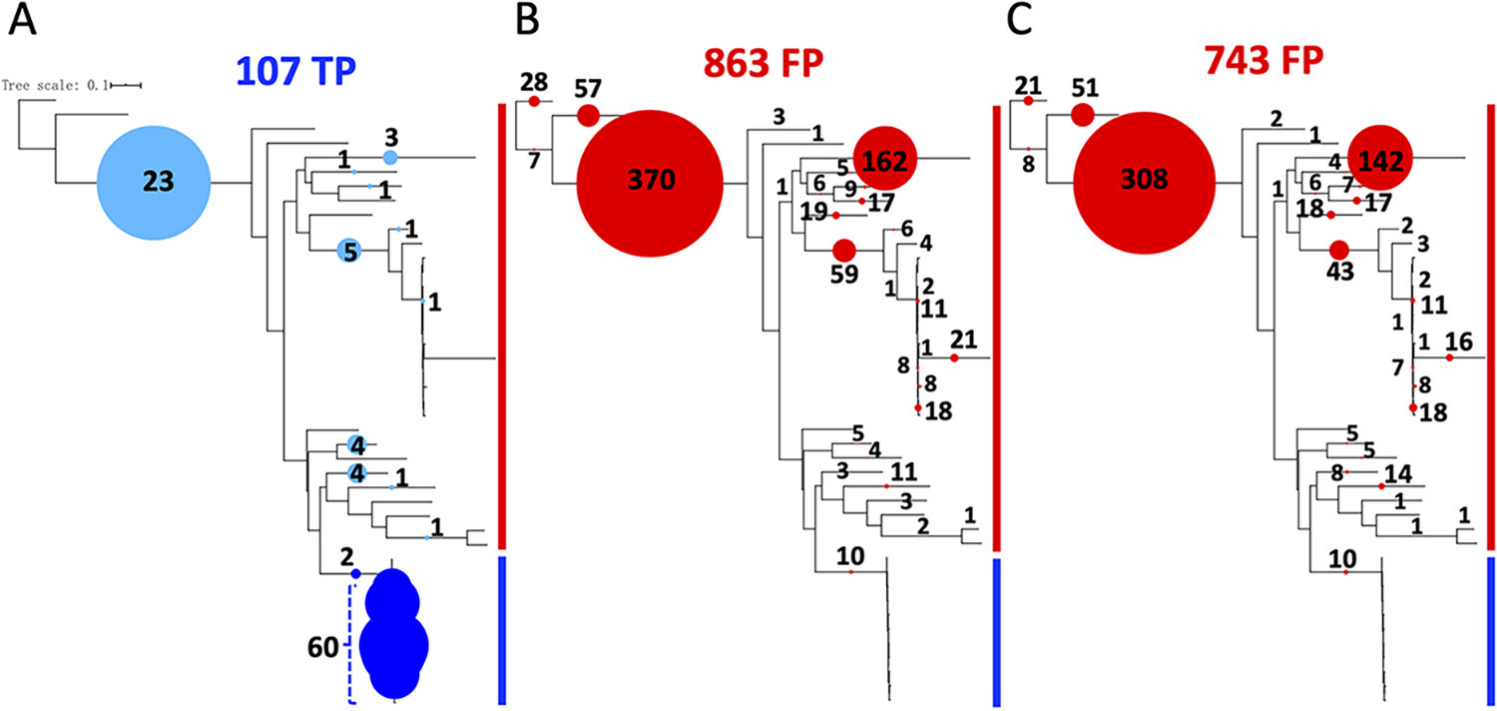
Phylogenetic placement of the *bla*_TEM_-carrying (true positive) and false-positive (from nontarget genes) reads identified in the 150-bp mock data set by different approaches. The bar to the right of each tree emphasizes clades made up of target (blue) and nontarget (red) reference proteins. The colored circles represent the numbers of reads that were placed on the corresponding branches. The number of reads that make up each circle is provided for direct comparison between the trees (the filled circles on the same tree are proportional to the number of reads and directly comparable to each other, but not directly comparable between trees). The positive references used to build the TEM ROCker model are in blue (reads originating from these sequences are target reads and are included in the blue circles as TP), and negative references used to build TEM ROCker model are in red (reads originating from these sequences are nontarget reads and are included in the red circles as FP). (A) Placement of 107 true positive reads identified in common by ROCker, BLASTx search (E value < 10^−5^), and searches with a custom-built TEM HMM (E value < 0.1). Note that several of these reads (in light blue) were (erroneously) placed to nontarget clades due to lack of phylogenetic signal (e.g., the reads sampled highly conserved regions between target and nontarget reference proteins) or sequence errors that confused the placement pipeline. The former reads were usually placed on ancestral nodes, while the latter were usually placed on terminal nodes. (B) Placement of 863 false-positive reads identified by BLASTx search with an E value of <10^−5^. (C) Placement of 743 false-positive reads identified by searches with a custom-built TEM HMM with an E value of <0.1. Note that a small number of these FP reads (*n* = 10) were (erroneously) placed on the clade made up of target sequences due to inadequate phylogenetic signal. The phylogenetic tree was constructed using maximum likelihood in RAxML version 7.7.2 with the GTRGAMMA model. Reads were placed on the phylogenetic tree as described in Materials and Methods. Note that no false negatives (i.e., *bla*_TEM_-carrying reads that were not detected by the tools) were observed by ROCker, BLASTx search (E value < 10^−5^), and searches with a custom-built TEM HMM (E value < 0.1) for this BL family.

In total, 43 amino acid reference sequences, including 11 positive TEM references and 32 negative references from other (nontarget) clades of class A BLs, such as SHV, LAP, TER, OKP, OXY, and CTX-M, were used to build a reference phylogenetic tree (Fig. 2). To further validate the results obtained by ROCker and the similarity searches and identify the exact sequence variant carried by the reads, the *bla*_TEM_-carrying reads identified by each tool in the mock data sets were placed in the reference phylogeny (Fig. 5). The Klebsiella pneumoniae carbapenemase (KPC) in the reference set of the ROCker model was excluded from the phylogenetic tree because no corresponding nucleotide sequence that was needed for nucleotide-based short-read placement was found in the EMBL database for this protein. The phylogenetic placement of the *bla*_TEM_-carrying reads (*n* = 107 in total) (Fig. 5A) in the 150-bp data set, which were identified as TP by all three approaches (i.e., ROCker, BLASTx search with an e-value of <10^−5^, and a custom-built TEM HMM with an e-value of <0.1), showed that a majority of the reads (about 60%) grouped with the TEM family, as expected. The remaining reads were associated with proteins closely related to TEM (75 to 78% amino acid identity) that clustered between the TEM and SHV families, such as the LAP, LEN, and OKP family, or were placed on more ancestral nodes (nondiscriminating) of the TEM and SHV lineages (about 20% of total remaining reads), presumably because these reads did not carry enough phylogenetic signal for placement to the tips of the reference tree, e.g., they carried domains shared between target (TEM) and nontarget (the remaining, non-TEM) protein sequences.

No FN reads were observed for any of three approaches for identifying TEM-carrying reads. Further, ROCker detected no FP (nontarget) reads, while the BLASTx search (e-value < 10^−5^) (Fig. 5B) and the custom-built TEM HMM (e-value < 0.1) (Fig. 5C) detected 863 and 743 FP reads, respectively. The phylogenetic placement patterns for these reads were consistent with the reads being FPs, i.e., the majority (89%) of the FP reads were placed in the SHV lineage and on the branches between the TEM and SHV (proteins closely related to TEM). A small number of the reads, about 10%, were assigned to the more divergent nontarget class A clades, i.e., OXY and CTX-M. Around 1% of the FP reads mapped to the TEM family due to the fact that the reads sampled a nondiscriminatory (highly conserved) part of the alignment (Fig. S5).

10.1128/mSystems.01281-21.7 FIG S5 Alignments of the 11 positive TEM protein references (top) and 32 negative protein references from other (nontarget in this case) clades of class A BLs (bottom). The amino acid reference sequences of the BLs were aligned using MAFFT version 7.407. The KPC in the reference set of the ROCker model was excluded from the phylogenetic tree because there was no corresponding nucleotide sequence for the gene encoding this protein in the EMBL database, which was needed for nucleotide-based short-read placement. Download FIG S5, PDF file, 0.4 MB. Copyright © 2022 Zhang et al. 2022 Zhang et al. https://creativecommons.org/licenses/by/4.0/ This content is distributed under the terms of the Creative Commons Attribution 4.0 International license.

Compared to the 150-bp data set, a lower number of TP reads using all methods (98 target reads) and a higher number of FP reads (1,396 by BLASTx and 1,287 by custom-built TEM HMM) were observed for the 250-bp data set (Fig. S6). The placement patterns for the TP (Fig. S6A) and FP (Fig. S6B and C) reads in the 250-bp data set were similar to those in the 150-bp data set, except that a slightly lower percentage (0.5%) of the FP reads were assigned to the TEM family, as expected for longer reads that are more discriminatory. Overall, the FP reads represented protein sequences that were closely related to the target sequences but had distinct functions, which the similarity searches (but not ROCker) were unable to distinguish from the target positive reads.

10.1128/mSystems.01281-21.8 FIG S6 Phylogenetic placement of the *bla*_TEM_-carrying and false-positive reads (from nontarget genes) identified in the 250-bp mock data set by different approaches. (A) Mapping of 98 true-positive reads identified in common by ROCker, BLASTx search (e-value < 10^−5^), and searches with a custom-built TEM HMM (e-value < 0.1). (B) Mapping of 1,396 false-positive reads identified by BLASTx search with an e-value of <10^−5^. (C) Mapping of 1,283 false-positive reads identified by searches with a custom-built TEM HMM with an e-value of <0.1. Circles in light blue denote reads that were placed onto nontarget genes or clades due to inadequate phylogenetic signal. The phylogenetic tree was constructed as described in Fig. 5. Download FIG S6, PDF file, 0.1 MB. Copyright © 2022 Zhang et al. 2022 Zhang et al. https://creativecommons.org/licenses/by/4.0/ This content is distributed under the terms of the Creative Commons Attribution 4.0 International license.

### (ii) Class D BL gene-carrying reads.

**FIG 6:**
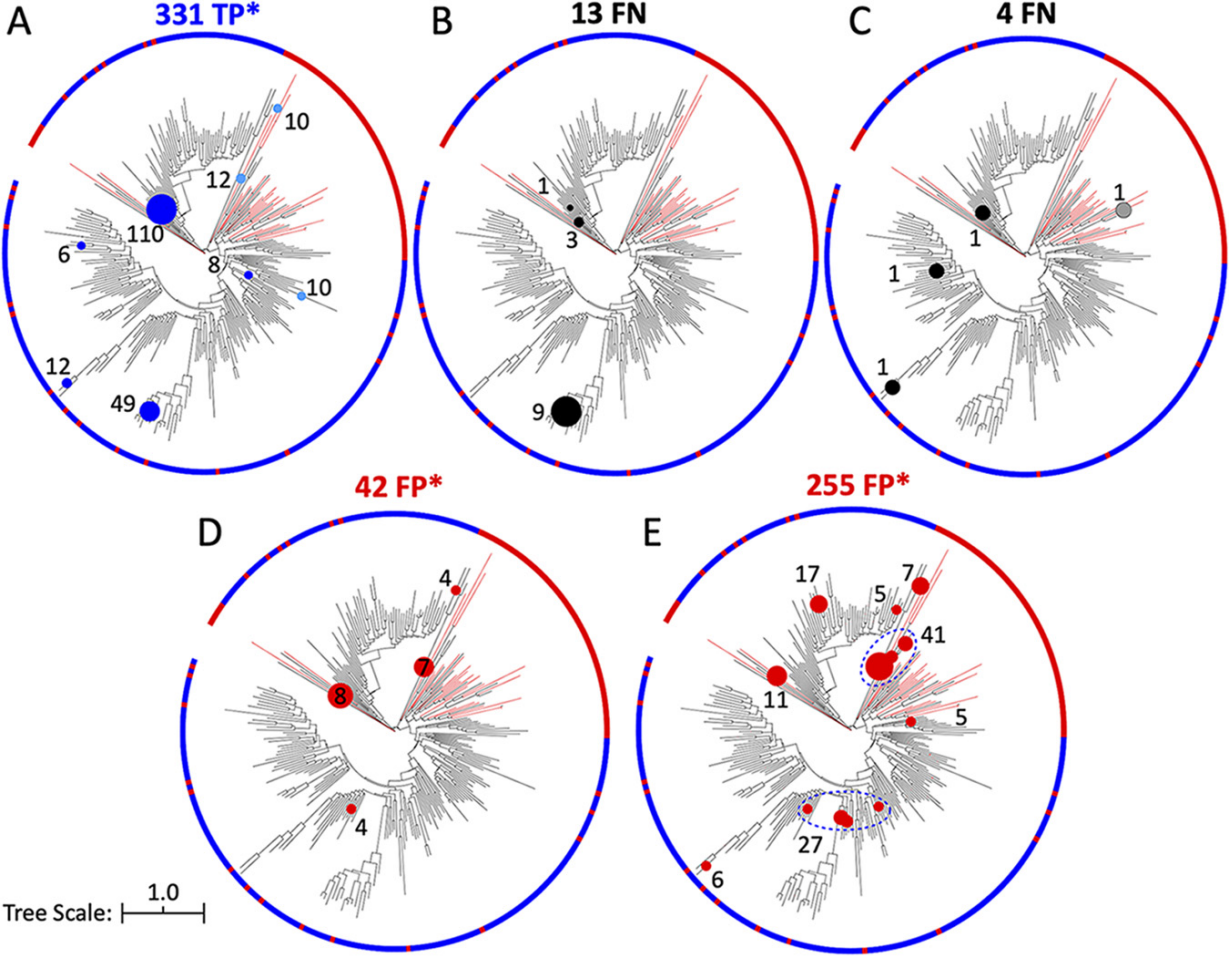
Phylogenetic placement of the class D BL gene-carrying reads (true positives and false negatives) and reads from nontarget genes (false-positive reads) identified in the 150-bp mock data set using different approaches. The same methods and parameters were used as described for Fig. 5, but a circular tree was chosen due to the higher number and more diverse sequences of the class D BLs; and in panels A, D, and E, the asterisk indicates that not all identified reads are shown for demonstration purposes (e.g., many clades recruited only a couple of reads). In addition, red branches indicate UniRef90 proteins that were included in the negative-reference set for ROCker model building (see also a more detailed class D BL phylogeny in Fig. S1). (A) True-positive reads (*n* = 331; 114 reads in 58 clades are not shown) detected by BLASTx search (E value < 10^−5^). (B) False-negative reads (*n* = 13) missed by ROCker (i.e., reads carrying class D BLs that ROCker did not detect). (C) False-negative reads (*n* = 4) missed by the custom-built class D BL HMM. (D) False-positive reads (*n* = 42; 19 reads in 15 clades are not shown) detected by BLASTx search (E value < 10^−5^). (E) False-positive reads (*n* = 255; 135 reads in 83 clades are not shown) detected by the custom-built class D BL HMM. As in Fig. 5, circles in light blue or gray denote reads that were placed on nontarget genes or clades due to inadequate phylogenetic signal.

The class D BL gene-carrying reads (150-bp data set) identified by ROCker, BLASTx search (e-value < 10^−5^), and the custom-built class D BL HMM (e-value < 0.1) were placed on the phylogenetic tree shown in Fig. 6A. A combined total of 159 (48%) of the TP reads were placed on the branches of the two verified class D BL references that were most closely related to the protein sequences used in the mock data (i.e., the two largest circles in Fig. 6A). The other 52% of TP reads were scattered around the tree on both target and nontarget branches and on more ancestral nodes, presumably because these reads originated from conserved regions that are difficult to distinguish between target and nontarget sequences.

10.1128/mSystems.01281-21.3 FIG S1 (A) Phylogenetic relationship of verified class A BLs (blue) with verified nontarget proteins (green) and other related proteins from the UniRef90 database. Red branches indicate proteins that were included in the negative-reference set for ROCker model building because they formed a branch with verified nontarget proteins; the remaining proteins (blue and black labels) were included in the positive-reference set. (B) Phylogenetic relationship of the verified metallo-β-lactamases (MBLs) with verified nontarget proteins (green) and other related proteins from the UniRef90 database. Blue, purple, and pink labels indicate functionally verified MBL subtypes 1, 2, and 3, respectively. Proteins with labels highlighted in yellow are functionally verified outgroups and were included in the negative-reference set for ROCker model building. Proteins in pink and blue branches were included in the positive-reference set. (C) Phylogenetic relationship of the verified class C BLs (blue) with verified nontarget proteins (green) and other related proteins from the UniRef90 database. Proteins with red labels were included in the negative-reference set for ROCker model building. Proteins with blue and black labels were included in the positive set. (D) Phylogenetic relationship of verified class D β-lactamases (BLs, blue) with verified, nontarget proteins (green) and other related proteins from the UniRef90 database. Proteins in red branches were included in the negative-reference set for ROCker model building because they form a branch with verified nontarget proteins. Proteins with orange labels were removed from the ROCker model building because they lack at least one of the three conserved domains of class D BLs. The remaining references were included in the positive set. Details are provided in Text S1. Download FIG S1, PDF file, 0.3 MB. Copyright © 2022 Zhang et al. 2022 Zhang et al. https://creativecommons.org/licenses/by/4.0/ This content is distributed under the terms of the Creative Commons Attribution 4.0 International license.

10.1128/mSystems.01281-21.1 TEXT S1 Supplemental methods, providing details on how reference protein sequences were determined for building the class A, B, and C ROCker models. Download Text S1, DOCX file, 0.03 MB. Copyright © 2022 Zhang et al. 2022 Zhang et al. https://creativecommons.org/licenses/by/4.0/ This content is distributed under the terms of the Creative Commons Attribution 4.0 International license.

Overall, a small number of the class D BL gene-carrying reads was missed in the 150-bp data set by these approaches, i.e., 13 FN reads out of the 331 target reads for ROCker (4% of the total class D BL gene-carrying reads) and 4 FN reads out of the 331 target reads for the custom-built HMM (1% of the total class D BL gene-carrying reads) (Fig. 6B and C). The BLASTx search at an e-value of 10^−5^ did not result in any FN reads. The majority of the FN reads (100% and 75% of the total FN reads for ROCker and custom-built HMM, respectively) were placed on target class D BLs. However, a single FN read in the custom-built HMM results (25% of the FN reads) mapped to a nontarget sequence. This read originated from a more conserved region of the gene that was difficult to distinguish between target and nontarget sequences. No FP (nontarget) reads were detected by ROCker, and the FP reads detected by BLASTx (e-value < 10^−5^; 42 reads) and custom-built HMM (255 reads) searches of the 150-bp data set were more dispersed around the tree than the TP or FN reads. In both cases, the highest number of FP reads placed on a single nontarget gene branch had only 19% (8 reads) of total FP reads for the BLASTx search (Fig. 6D) and 11% (27 reads) of total FP reads for the custom-built HMM (Fig. 6E) placed to distinct nontarget genes. Similar placement patterns of the TP, FN, and FP reads were obtained for the 250-bp mock data set (Fig. S7), except that a slightly lower number of TP reads (331 versus 316 targets for the 150-bp and 250-bp data sets, respectively) and a higher number of FP reads detected by BLASTx (42 versus 113 FP reads for the 150-bp and 250-bp data sets, respectively) and the custom built HMM (255 versus 540 FP reads for the 150-bp and 250-bp data sets, respectively) were observed.

10.1128/mSystems.01281-21.9 FIG S7 Phylogenetic placement of the class D BL gene-carrying and false-positive reads (from nontarget genes) identified in the 250-bp MOCK data set by different approaches. (A) True-positive reads (*n* = 316; 47 reads in 26 clades are not shown) detected by BLASTx search (e-value < 10^−5^). (B) False-negative reads (*n* = 2) missed by ROCker (i.e., reads carrying class D BLs that ROCker did not detect). (C) False-positive reads (*n* = 113; 27 reads in 22 clades are not shown) detected by BLASTx search (e-value < 10^−5^). (D) False-positive reads (*n* = 540; 189 reads in 89 clades are not shown) detected by the custom-built class D BL HMM. Circles in light blue denote reads that were placed on nontarget genes or clades due to inadequate phylogenetic signal. The phylogenetic tree was constructed as described in the legend to Fig. 6. Download FIG S7, PDF file, 0.3 MB. Copyright © 2022 Zhang et al. 2022 Zhang et al. https://creativecommons.org/licenses/by/4.0/ This content is distributed under the terms of the Creative Commons Attribution 4.0 International license.

### Comparison to other bioinformatics tools.

We also evaluated other tools for the detection of target ARGs in shotgun data, such as DeepARG (12), ShortBRED (26), and AMRFinder (https://ncbi.nlm.nih.gov/pathogens/antimicrobial-resistance/AMRFinder/). It should be noted that these tools were designed with slightly different goals in mind. For instance, DeepARG is designed for finding novel, deep-branching (divergent) homologs to known ARG. ShortBRED is optimized for accurate functional profiling of metagenomic samples by focusing on unique motifs or segments of the targeted protein; hence, ShortBRED does not usually capture reads representing shared motifs with other nontargeted proteins. Similarly, AMRFinder is tuned for complete sequences, not fragments carried by individual metagenomic reads. Accordingly, the comparison of ROCker to these other tools was performed mostly to further highlight the distinctive strengths of the ROCker models. Consistent with our expectations, we found that the combined results from the four ROCker models covering all known classes of BLs (classes A, B, C, and D) had higher precision (over 70 times lower FPR) and recall (over 5 times lower FNR) than DeepARG or AMRFinder. Note also that the trends observed with AMRFinder were similar to those of BLASTX reported above, consistent with our expectations, since AMRFinder is essentially a BLASTX search (with tuned parameters). Likewise, ROCker had substantially better recall and precision than ShortBRED (over 30 times lower FNR and over 50 times lower FPR for both the 150-bp and 250-bp data sets) (Fig. S8). Taken together, these results further corroborated the advantages of ROCker models for identifying short reads carrying a specific protein of interest (target).

10.1128/mSystems.01281-21.10 FIG S8 Performance of the ROCker model on 150-bp (A) and 250-bp (B) mock data sets compared with alternative methods. Alternative methods included DeepARG, ShortBRED, AMRFinder, and BLASTx search using a fixed e-value (10^−3^, 10^−5^, 10^−10^, 10^−15^, and 10^−20^). The false-positive rate [FP/(TP + FP)], the false-negative rate [FN/(TP + FN)], and the F1 score [2 × precision × recall/(precision + recall)] are shown. Download FIG S8, PDF file, 0.1 MB. Copyright © 2022 Zhang et al. 2022 Zhang et al. https://creativecommons.org/licenses/by/4.0/ This content is distributed under the terms of the Creative Commons Attribution 4.0 International license.

### Technical challenges and tips when using a ROCker model. (i) Similarity search tool to use.

The first version of the TEM ROCker model was built using the default settings of ROCker; i.e., the reference protein sequences provided were queried against the simulated shotgun data sets using BLASTx (27) with the e-value set to 0.01. Only the best matches for each read of the training data sets were considered further to compile and plot the model using the script BlastTab.best_hit_sorted.pl (Enveomics Collection [28]). Based on the plot of the TEM ROCker model (i.e., validation plot before querying the real metagenome or mock data sets), the relatively low sensitivity (85.3%) of the model was due to an unexpected drop in the bit score of a highly conserved region of the target sequences (Fig. S2C). After manually inspection of the BLASTp output of the target sequences, it became clear that these reads were assigned a lower bit score than expected due to a BLAST filter for low-complexity sequences. To avoid this problem, either BLASTx with “-seg no” or a DIAMOND search can be used instead of the default BLASTx settings. A comparison of the plot statistics revealed that using DIAMOND with settings of “min score” of 20 and “sensitive” (24), among several settings evaluated, had the highest sensitivity (98.8%) compared to the default BLASTx (85.3%) or BLASTx with “-seg no” settings (95.6%) and was significantly faster, as previously demonstrated (29). Thus, DIAMOND is currently recommended for searching reads against a reference alignment.

### (ii) Trimming the alignment.

While examining the plot of the class D BL ROCker model, we noticed that the first 100 amino acid residues of the alignment contained a region with low conservation among the target sequences (i.e., >50% of the alignment columns had gaps and/or low amino acid identity) that was similar to the conservation levels between target and nontarget sequences and thus not useful for diagnostic purposes (i.e., it resulted in frequent false-positive matches). Trimming the first 100 residues from the alignment for the 150-bp class D BL model improved sensitivity by ~0.2% (Fig. 1B and Fig. S4C). Trimming the last 440 residues from the MBL models improved sensitivity by 61.5% (Fig. S3C and D); thus, removing such nondiscriminatory regions of the alignment could improve model performance. The length of such nondiscriminatory regions of the protein sequence relative to the read length should also be considered in making a decision about whether to trim the alignment (e.g., if the length of such regions is too short compared to the length of the target sequences or the read length, such as half the read length or shorter, then trimming is not as useful).

10.1128/mSystems.01281-21.5 FIG S3 (A and B) Plot of the untrimmed (A) and trimmed (B) 150-bp ROCker model of class A BLs using DIAMOND search. These models were built using the pipeline described in the text, except that the first 150 and last 30 columns of the alignment were removed (i.e., trimmed) to improve model performance and reduce the number of false positives, since this region was highly variable among the positive sequence set. The sensitivity, specificity, and accuracy were 98.5%, 99.9%, and 99.9% for the untrimmed and 98.1%, 99.9%, and 99.9% for the trimmed class A BL ROCker models, respectively. (C and D) Plot of the untrimmed (C) and trimmed (D) 150-bp ROCker model of metallo-β-lactamase (MBL) subtypes 1, 2, and 3 combined using a DIAMOND search. The first 80 and last 440 columns of the alignment were removed. The sensitivity, specificity, and accuracy were 36.3%, 99.5%, and 96.7% for the untrimmed and 97.8%, 99.1%, and 99.1% for the trimmed MBL ROCker models, respectively. (E and F) Plot of the 150-bp ROCker model of MBL subtypes 1 and 2 combined (E) and subtype 3 (F) using a DIAMOND search. The first 91 and last 101 columns of the alignment were removed; for the subtype 3 model, the first 107 and last 445 columns of the alignment were removed. The sensitivity, specificity, and accuracy were 99.0%, 99.8%, and 99.7% for the S1/S2 model and 94.3%, 99.8%, and 99.6% for the S3 model, respectively. Download FIG S3, PDF file, 1.6 MB. Copyright © 2022 Zhang et al. 2022 Zhang et al. https://creativecommons.org/licenses/by/4.0/ This content is distributed under the terms of the Creative Commons Attribution 4.0 International license.

10.1128/mSystems.01281-21.6 FIG S4 (A and B) Plot of the untrimmed (A) and trimmed (B) 150-bp ROCker model of class C BL using BLASTx search with default settings. The first 40 columns of the alignment were removed. The sensitivity, specificity, and accuracy were 98.1%, 99.9%, and 99.9% for the untrimmed and 98.1%, 99.9%, and 99.9% for the trimmed class C BL ROCker models, respectively. (C and D) Plot of the original, untrimmed 150-bp (C) and 250-bp (D) ROCker models of class D BL using a DIAMOND search. The sensitivity, specificity, and accuracy were 99.0%, 99.8%, and 99.8% for the 150-bp model and 98.4%, 100%, and 100% for the 250-bp model, respectively. Download FIG S4, PDF file, 1.3 MB. Copyright © 2022 Zhang et al. 2022 Zhang et al. https://creativecommons.org/licenses/by/4.0/ This content is distributed under the terms of the Creative Commons Attribution 4.0 International license.

### (iii) Implementing a length correction for reads shorter than expected in ROCker.

The alignment statistic used to discriminate true from false-positive matches in ROCker is the bit score, which is sensitive to alignment length. Consequently, large variations in read lengths may result in an unexpectedly high FNR (for shorter reads) and, occasionally, a high FPR (for longer reads when closely related nontarget proteins are present). In order to account for such length variations, we implemented the option -L in ROCker search and filter actions (v1.5.2+), which corrects the bit scores according to the ratio of expected to observed query read length with an additional penalty; i.e., if a read is half of the expected read length, its observed bit score will be multiplied by a factor of 2 to account for the shorter length minus a penalty to avoid overinflating results from reads shorter than expected. The penalty can be variable, which assumes that the probability of observing a proportionally higher bit score if the reads were of the expected length decays with the difference between expected and observed length until the maximum length correction (-M), termed the triangle method in the documentation of ROCker. Alternatively, a fixed fraction of the difference can be used as the penalty (-P). In our tests, we observed the best results using the -L option together with a fraction penalty (-P) of 0.5. However, the data sets used in this study do not exhibit large read length variation, and therefore, we did not apply this correction. The correction is recommended for data sets with substantial variation in read length, such as when 20 to 30% or more of the reads differ in length by more than 25%.

## DISCUSSION

While similarity or HMM searches are commonly used to functionally annotate short-read metagenomes (27, 30–32), deciding the optimal e-value threshold for the searches remains a challenge (33, 34). In addition to the well-appreciated factors, such as the size of the database and the length of the query sequence used, the sequence diversity of the target sequences is also important in determining the optimal e-value threshold. A particularly good example of the difficulties associated with searches for functional annotation is epitomized by the β-lactamases, which include families of proteins with high sequence diversity, as well as families with little to no sequence diversity. For instance, for the class D BLs, which have a higher level of diversity, similarity searches with a fixed e-value at 10^−10^ showed performance comparable to that of the ROCker model, with virtually no FP or FN detected in the mock data sets used to test these calling methods (Data Set S1, sheet 5). In fact, ROCker missed a small number (4%) of target reads (FN) that BLASTx with an e-value at 10^−10^ or 10^−20^ did not miss, presumably because these e-value thresholds happen to be ideal for separating target from nontarget reads in this (but not necessarily any other) mock data set. However, for TEM (family-level BLs), which are highly similar among themselves, using the same e-value threshold at 10^−10^ for similarity searching resulted in a large number of FP reads (434 and 1,055 for the 150-bp and 250-bp data sets, respectively); even a more stringent e-value threshold still resulted in a large number of FP reads, especially for the longer-read data set, i.e., FPR of 61% for the 250-bp data set (Data Set S1, sheet 4). It is also important to note that with a more stringent e-value threshold, the decrease in FPR is accompanied by a proportional increase of FNR for BLAST- or HMM-based approaches (Fig. 3 and 4), and the optimal e-value threshold to employ remains typically elusive in most studies due to the lack of mock data sets and/or an *a priori* appreciation of the diversity of the target sequences in the metagenomic data set of interest.

The analysis of mock data sets with BL gene-carrying reads showed that ROCker can effectively sidestep these limitations because it is based on a position-specific calculation of the most discriminant bit score thresholds that do not depend on data set size or target length (24). Accordingly, ROCker provided much higher F1 scores than using similarity or HMM searches with a fixed e-value threshold of 0.1 or below for the same purposes (e.g., Fig. 3 and 4). Thus, ROCker is a particularly powerful approach for proteins such as BLs due to the various diversity levels among subfamilies and different sequence lengths (8, 35–37). It is also important to note that the ROCker-based pipeline described above can be applied to amplicon sequencing data, such as those from high-throughput PCR platforms, to evaluate nonspecific primer amplification of nontarget sequences and/or type target sequences against a reference phylogeny of the target (38, 39).

Maximum-likelihood placement of reads against the reference phylogenetic tree can be a highly accurate method for identification and typing of reads carrying the gene of interest (target) at the individual allele level, in general. However, this method is not practical for processing a large number of reads (in the thousands or millions) in a reasonable time. Hence, we employed read placement here, which is computationally tractable as an additional step to the ROCker-based filtering of reads. Phylogenetic placement of the reads further validated the results of ROCker and the high frequency of TP calls; i.e., target reads were mapped to the branches expected based on the reference sequence from which the reads originated (Fig. 5A and 6A). However, in several cases, the *bla*_TEM_-carrying (Fig. 5A) and nontarget-carrying reads (FP reads) (Fig. 5B and C) were placed on the same branches, rendering it challenging to distinguish between TP and FP reads based on the phylogenetic approach. These reads mostly originated from a highly identical region shared between TEM and closely related (non-TEM) proteins, such as proteins from the TEM/SHV clade within class A BLs (8) (Fig. S5). ROCker was able to identify these regions of the target protein at the modeling compilation step by increasing the bit score threshold of the corresponding windows and thus did not make these FP calls. Thus, ROCker can be advantageous in such cases even over phylogeny-based approaches in terms of not overcalling FP matches in regions of high sequence similarity (low phylogenetic signal) between target and nontarget sequences. The approach presented here, i.e., using ROCker to filter matching reads combined with phylogenetic placement of the identified reads against the tree of the positive- and negative-reference sequences, takes advantage of the strengths of these two methods in a complementary fashion.

The ROCker models for the four known classes of BLs, i.e., class A (Fig. S3B), B (Fig. S3D), C (Fig. S4B), and D (Fig. 1B and Fig. S2B) BLs, and also for BLs from the phylogenetically narrow TEM family (Fig. 1A and Fig. S2A) are available through http://enve-omics.ce.gatech.edu/rocker/models#fam:BLA for online analysis of short-read data sets provided by external users. The TEM and class D BL models described herein represent detailed examples of how to build and test ROCker models for highly related proteins and highly diverse proteins, respectively. Selecting the appropriate positive-reference sequences and, when needed, negative references and manually verifying them constitute the key and most time-consuming step in building robust ROCker models, and we provide examples of how to perform this step and caveats for what to avoid. In addition, we are committed to keep supporting the above-mentioned website and adding new models for additional ARG families as these become available; external users are also welcome to contact us for development of custom ROCker models.

In conclusion, our work demonstrates the reliable detection and typing of short read sequences carrying BLs by ROCker models and the higher accuracy of this approach compared to alternatives for the same purpose and type of data. Therefore, application of these BL ROCker models on metagenomics, metatranscriptomics, or high-throughput PCR gene amplicon data sets from various environments should enable the reliable detection and quantification of BL variants encoded by environmental or clinical isolates and microbiomes, while avoiding high-frequency false-positive calls. Furthermore, the curated ROCker models and reference BL sequences available through our web server should facilitate the development of new models for additional (phylogenetically narrow) BL families as well as non-BL ARGs. Therefore, the ROCker models presented here substantially expand the toolbox for monitoring antibiotic resistance in clinical or environmental samples and assessing public health risk (40).

## MATERIALS AND METHODS

### Overview of the ROCker workflow to identify BLs in short read data.

For a detailed description of ROCker, see reference 24. Briefly, during the target model building process, a user-provided list of UniProt IDs of positive (target) and negative (nontarget) reference proteins are used to identify the corresponding whole-genome sequences encoding these proteins (for how to select appropriate positive-reference sequences, see below). The genome nucleotide sequences are downloaded and Illumina-like short reads are simulated from the sequences using Grinder (25) in order to generate short-read training data sets, while keeping track of which reads carry the target reference genes of interest (target reads; based on UniProt IDs provided) and which reads represent the rest of the genome (nontarget reads). The positive full-length references are translated and protein sequences aligned using ClustalΩ (41), and the resulting alignment is used as the database to query both the target and nontarget simulated short-read data sets, using BLASTx (27) or DIAMOND (29). The ROCker pipeline then calculates the most discriminant bit score in short windows across the alignment of the reference sequences that best differentiate target versus nontarget reads using ROC curves and produces a plot with summarizing statistics of the accuracy of the model based on the training data set. An example of the plot that also includes the graphical representation of the alignments, the calculated bit score thresholds, and the matches obtained is provided in Fig. 1. In general, regions of the protein reference alignment with higher amino acid conservation will have higher bit score thresholds than more divergent regions; high bit scores are also expected when nontarget sequences share domains or motifs with target sequences based on the training data sets. The ROCker model is composed of the estimated best bit score thresholds for short reads along the positive protein reference alignment and can subsequently be used to filter the search results of a real (or mock) metagenomic data set against the same reference sequences.

### Assessing the total diversity of BLs and building a reference phylogeny for validating ROCker results and alternative methods.

It is important to note that the most critical step in building a ROCker model, and the only step that is not automated, is the choice of the target (positive) versus nontarget (negative) reference sequences (24). Hence, in the following sections, we describe our work with TEM and class D BLs as examples of the procedures that were followed for developing ROCker models for all classes of BLs to avoid redundancy, focusing on the choice of positive/negative sequences. These models and their results are provided in the supplemental material.

To build a list of TEM and class D BL positive references for input into the ROCker model building workflow, we first verified reference sequences of TEM-like variants and class D BL-like variants collected from ResFinder (14) and the Lactamase Engineering Database (part of the BioCatNet) (42–44) in October 2018. Verified sequences included those that were previously shown to inactivate the corresponding β-lactam based on genetic and/or phenotypic tests or showed conservation of functional domains and high sequence identity to such experimentally determined lactamases based on visual inspection of the alignments. These verified sequences were dereplicated using cd-hit version 4.6.1 (45, 46) at 90% amino acid identity, which resulted in 1 and 39 representative sequences for TEM-like variants and class D BL-like variants, respectively. These reference sequences were then used to search against larger public databases in order to identify additional homologs and cover the total diversity of the proteins currently available. For instance, the experimentally verified TEM-like variants were searched against the Swiss-Prot and TrEMBL (UniRef90; downloaded October 2018) databases using BLASTp (BLAST+ version 2.2.28) (27) with a cutoff coverage of the reference protein length of >90%. In order to capture more recently deposited sequences and any sequences missed by Swiss-Prot and TrEMBL, we also checked for additional, unique matches in the NCBI’s NR database.

Finally, all matched reference sequences were dereplicated at 90% amino acid identity using cd-hit version 4.6.1, and one representative per resulting cluster was aligned with the verified references using MAFFT version 7.407 (47). See the next section for how positive (target) and negative (nontarget) sequences were identified specifically for each target protein family. To describe the phylogenetic diversity of TEM-like variants as well as to map short reads on the phylogeny in order to validate the findings of the different tools evaluated (e.g., detect false positives), the MAFFT alignment was used to build a maximum-likelihood tree using RAxML version 7.7.2 (48) with the GTRGAMMA model (Fig. 1). Reads identified by the evaluated tools were mapped to this reference phylogeny as described below. The same approach was used for the class D BL sequences, except that the TrEMBL sequences were dereplicated at 50% amino acid identity due to the higher level of diversity among members of this class.

### Determining the reference protein sequences for building the ROCker model.

Protein sequences of TEM were obtained from the UniProt database and were aligned and manually inspected for the presence of the known functional motifs of TEM, i.e., R^65^FxxxS^70^xxK, S^130^DN, and K[TS]G (Brackets denote that either amino acid shown could be in that second position) (8). To more comprehensively cover the diversity of this family, TEM from different taxa were included in the positive-reference sequences (Data Set S1, sheet 1) for building the ROCker model. In order to improve the performance of the model, a second list of negative (i.e., nontarget) references that represented evolutionarily related antimicrobial resistance proteins with different functions, such as SHV (sulfhydryl variable β-lactamase), OXY (oxytoca β-lactamase), CTX-M (cefotaxime β-lactamase), and KPC (Klebsiella pneumoniae carbapenemase) (Fig. 2 and Data Set S1, sheet 1) were also included, as suggested previously, for increased accuracy when related (nontarget) proteins are expected to be present in the query data sets (24). The ROCker model was built using a DIAMOND search (29) with default settings (24).

More specifically, because the TEM-like variants share high sequence identity (98 to 99% amino acid identity) to each other (low intrafamily diversity), only sequences for a few TEM-like representatives are necessary for the positive-reference set. In total, 11 TEM protein sequences from different organisms, including Klebsiella oxytoca, Neisseria gonorrhoeae, Escherichia coli, Salmonella enterica serotype Typhimurium, Kluyvera georgiana and Proteus mirabilis, were included as positive-reference sequences for model building (Data Set S1, sheet 1). Due to the functional domains BLs share with nontarget proteins, such as lactam-binding (but nonhydrolyzing) proteins, and in order to obtain high, family-level resolution with the ROCker model, we included representatives of nontarget proteins as negative-reference sequences during model building, as suggested previously (24). Reads from nontarget gene sequences carrying the functional domains shared with the target sequences will provide relatively high bit scores, and the use of negative-reference sequences lowers the probability of these reads being mistaken for positive (target) reads during the testing step. The negative references (33 sequences in total) (Data Set S1, sheet 1) were selected from two sets of sequences: the closely related SHV family (~65% amino acid identity to TEM), and nonannotated protein sequences recovered from UniProt that clustered between the TEM and SHV (60 to 80% amino acid identity to TEM), as shown on the phylogenetic tree (Fig. 2). Protein sequences from the non-TEM families of class A BLs (8), including one OXY, one CTX-M, and one KPC, were also included as negative references for more comprehensive coverage of related, nontarget sequences. In general, for robust ROCker models, it is important for the negative-reference sequence set to evenly cover the phylogenetic diversity of related, nontarget proteins (if such proteins exist) in order to capture well the range of bit scores provided by short reads originating from such nontarget sequences when searched against the reference positive sequences relative to target reads.

For class D BLs, the alignment of reference sequences was manually verified for the two conserved serine residues (i.e., S^70^ and S^130^) and the K[TS]G domain (8), as described above for conserved functional motifs of TEM. The nontarget references, such as DacA (d-alanine transpeptidase), MecR1 (methicillin resistance protein), and BlaR1 (penicillin-binding regulatory protein), were also manually verified to have these conserved serine residues and K[TS]G domains, because they are motifs of the penicillin binding superfamily that includes proteins not conferring antibiotic resistance. As these domains are essential for proper enzyme folding and function but are not specific to antibiotic resistance functions, a phylogenetic approach was used to select the positive and negative references for ROCker model building (see Data Set S1, sheet 2, for files used and Fig. S1D). Sequences that formed a clade with nontarget reference sequences were included in the negative set. Sequences that were missing at least one of the three functional residues/domains were removed from model building under the assumption that these may be pseudogenes or homologs that have evolved to have different substrate specificities. The positive sequences for ROCker model building included representatives that had the conserved residues/domains and formed a clade with the proteins verified to be class D BLs (which were also included in model building) (Data Set S1, sheet 2).

More specifically, a total of 269 class D BL references were collected from the ResFinder database (downloaded October 2018), resulting in 39 sequences when dereplicated at 90% amino acid identity. These sequences showed high sequence diversity among them (e.g., often showing only ~30 to 40% amino acid identity). One representative sequence from each of the two major clades in the class D BL tree (Fig. S1D) was used as a query sequence against the Swiss-Prot database to find the most similar, experimentally verified non-BL proteins to use as outgroups and as negative references for the model building (described in Data Set S1, sheet 2). The 39 verified class D BL sequences were also queried against the UniRef90 database to find additional class D BLs and cover the described diversity of this class, which resulted in a total of 1,148 matches with query coverage of the target protein length of >90%. These matches were further dereplicated at 50% identity into 227 protein sequences, and the alignments were manually inspected for the conserved domains as described above. A total of 29 sequences lacked at least 1 of the 3 domains and thus were excluded from model building. Another 44 sequences were excluded because there were no corresponding nucleotide sequences for them in the EMBL database, and these were needed for nucleotide-based short-read placement. Finally, 24 proteins formed clades with the verified outgroups and were used as negative references, while 130 sequences were used as positive sequences for the model building. The list of reference sequences for the class D BL model is provided in Data Set S1, sheet 2.

ROCker relies on UniProt protein sequence identifiers to provide the list of the positive and negative-reference proteins (UniProt IDs) for model training. If users wish to use positive or negative protein sequences with no UniProt ID, this option is currently not supported. However, if a UniProt homolog with sufficient sequence identity to such sequences can be identified (>80% identity and complete alignment or overlap are recommended for most cases), the UniProt homolog could be used instead.

### ROCker models for other BLs.

ROCker models for class A, B, and C BLs were also developed using the approach described above for the class D BLs and are described in detail in the legends of Fig. S1 to S4. All models are available on our website (http://enve-omics.ce.gatech.edu/rocker/models#fam:BLA). In addition, more ROCker models can be found on our website, and readers may contact us if they wish to upload and make available their own ROCker models for general use.

### Simulated mock data sets for testing ROCker models and alternative methods.

In order to generate mock data sets of known composition, target BL and nontarget gene sequences were collected together with the genome sequences that carried these genes (Data Set S1, sheet 3). The gene sequences were selected to include high-confidence target or nontarget sequences that were not used in the construction of the ROCker or hidden Markov models (HMMs) (see below) to better challenge these tools. For each gene, the source genome was recovered through the EBI web services, and metagenomes were simulated from all whole-genome sequences using Grinder (25) with the options -dc -~*NnKkMmRrYySsWwBbVvHhDdXx -md uniform 0.1 -mr 95 5 -rd ReadLength uniform 5, where ReadLength corresponds to 150 or 250 bp, depending on the tested read length. Each simulation was repeated five times. The resulting reads derived from target or nontarget genes were tagged accordingly and concatenated, resulting in two mock data sets: one for 150-bp and one for 250-bp reads. These mock data sets are available online at http://enve-omics.ce.gatech.edu/data/rocker-bla. The mock data sets were used to assess the accuracy of ROCker relative to that of alternative methods for BL detection such as BLASTx searches (27) using fixed e-values (10^−2^, 10^−5^, 10^−10^, 10^−20^, and 10^−30^) or hmmsearch using HMMer version 3.1 (49) with an e-value of <0.1. The BL HMMs were downloaded from the FunGene (50) and Pfam databases (51) or custom built (available at http://enve-omics.ce.gatech.edu/data/rocker-bla) using the same positive-reference set that was used to build the ROCker BL model to enable direct comparisons.

### Phylogenetic placement of target BL gene-carrying reads.

To further validate reads from the mock data sets identified by ROCker and alternative methods, we placed the reads identified by each method onto a phylogenetic reference tree as follows. The amino acid sequences of full-length reference BLs were aligned using MAFFT version 7.407 (47) with default parameters and were then used as a guide to align their corresponding nucleotide reference sequences with the EMBOSS tranalign tool (52). The identified BL gene-carrying reads were then added to the nucleotide reference alignments using MAFFT version 7.407 (47) with the “addfragments” option and were placed in the corresponding phylogenetic tree (amino acid reference BL sequences) using RAxML with the -f v option. The placement of the target BL gene-carrying reads was visualized in iTOL (53) after processing of the resulting visualization jplace file with JPlace.to_iToL.rb from the Enveomics Collection (28).

### Data availability.

The ROCker models for class A, B, C, and D BLs and for the TEM family are available at http://enve-omics.ce.gatech.edu/rocker/models#fam:BLA. Further, users can upload their shotgun metagenomic or amplicon reads to the website and identify the BL gene-carrying reads using these models following the “Search online” link. An implementation to build ROCker models online is also available as part of the website as well as a Singularity container for local implementations of the complete ROCker pipeline. The 150-bp and 250-bp mock data sets are available online at http://enve-omics.ce.gatech.edu/data/rocker-bla.
