# Supplementary figures and images for: ROCker Models for Reliable Detection and Typing of Short-Read Sequences Carrying β-Lactamase Genes
Source: mSystems. 2022 May 31;7(3):e01281-21. doi: 10.1128/msystems.01281-21 (PMC9238382; doi:10.1128/msystems.01281-21)

**A**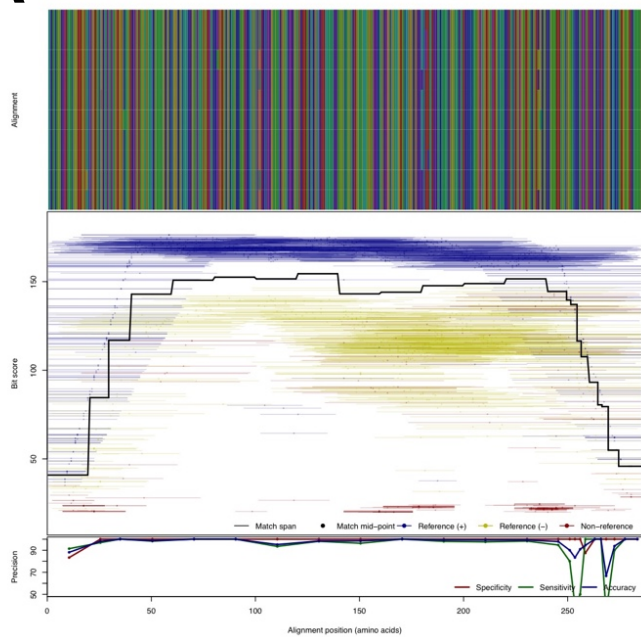**B**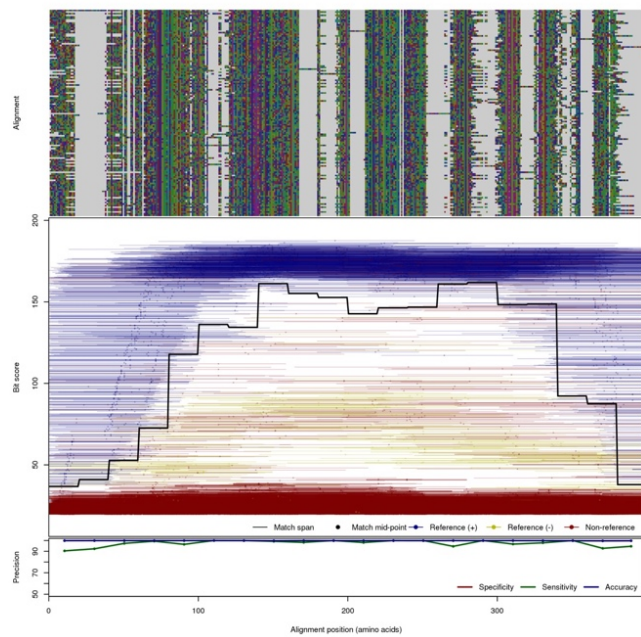**C**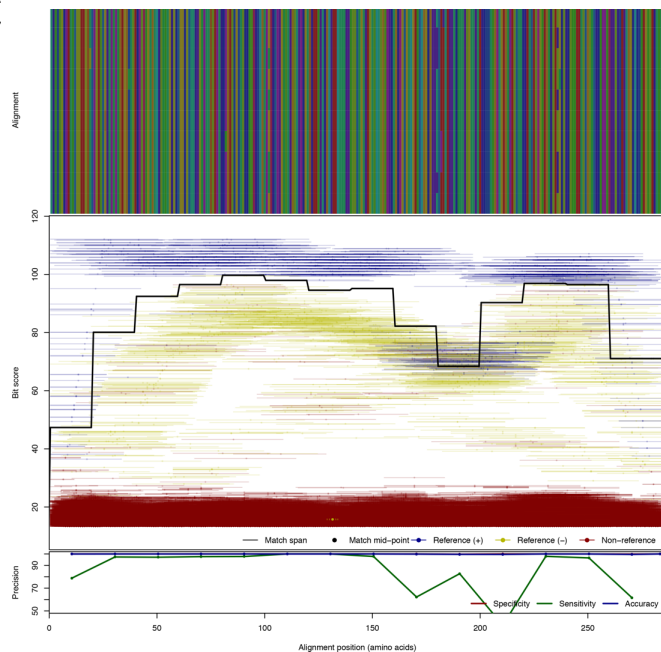

Supplement: FIG S2 [file msystems.01281-21-s0002.pdf]

**98 TP**

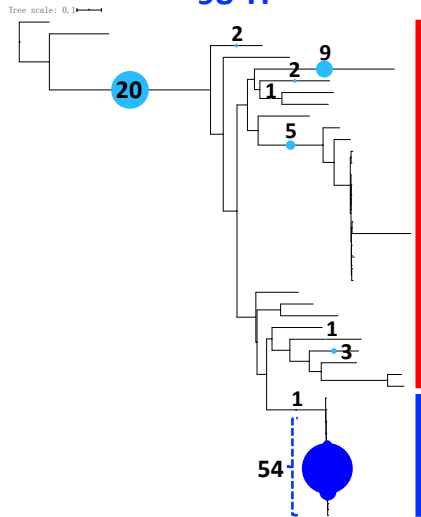

B

**1396 FP**

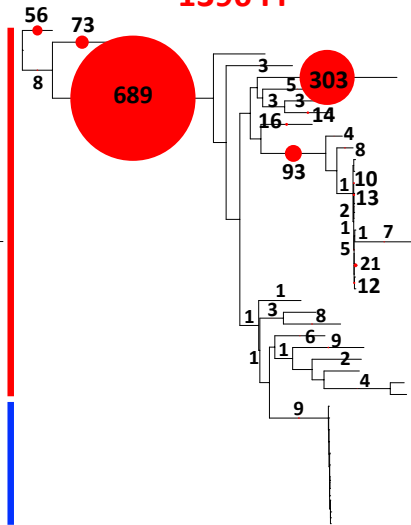

C

**1283 FP**

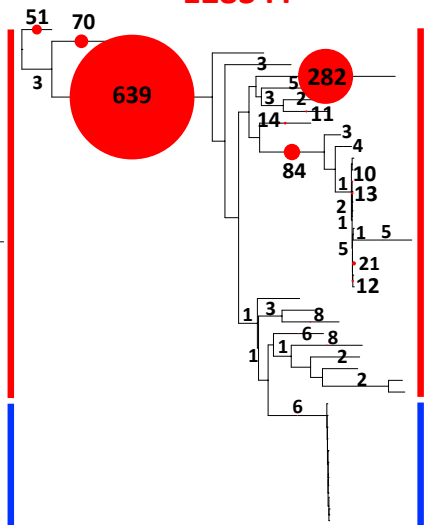

Supplement: FIG S6 [file msystems.01281-21-s0006.pdf]

**A**

Tree scale: 0.1

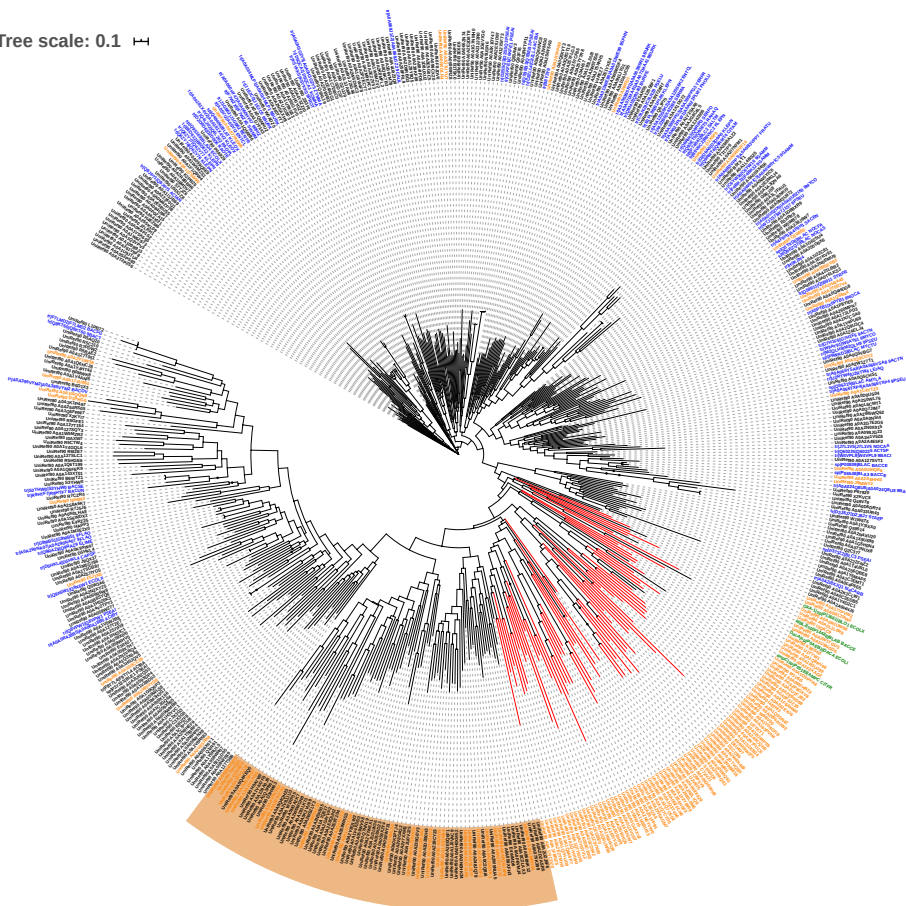**B**

Tree scale: 1

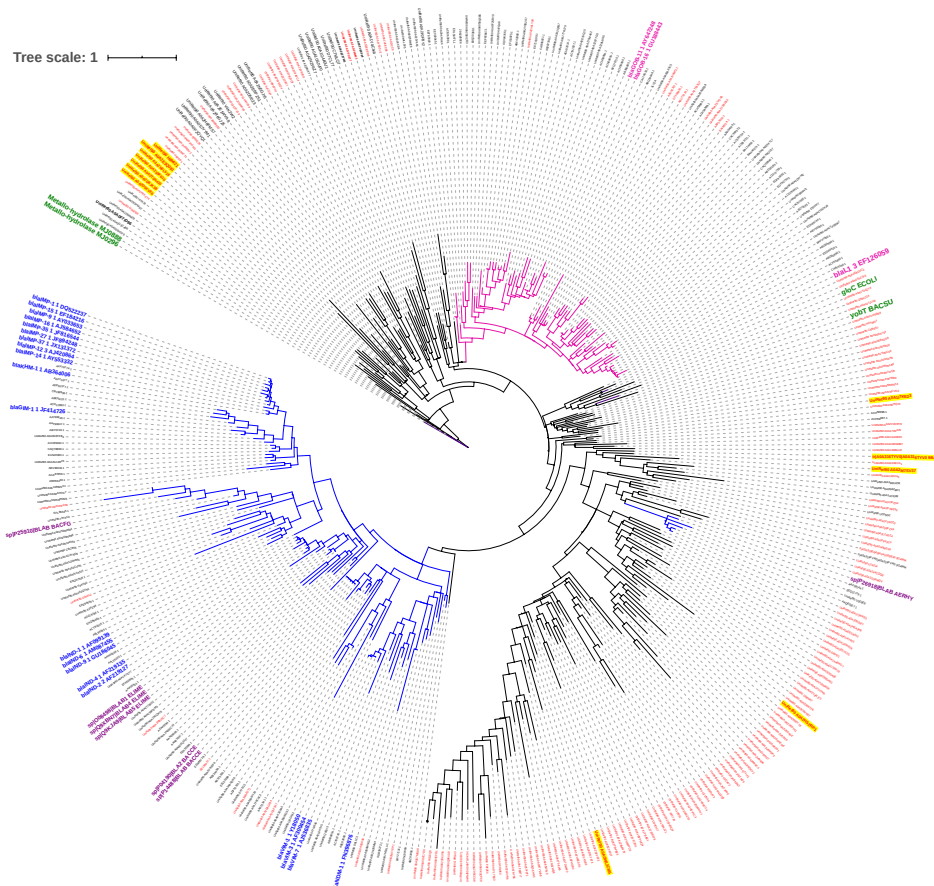**C**

Tree scale: 1

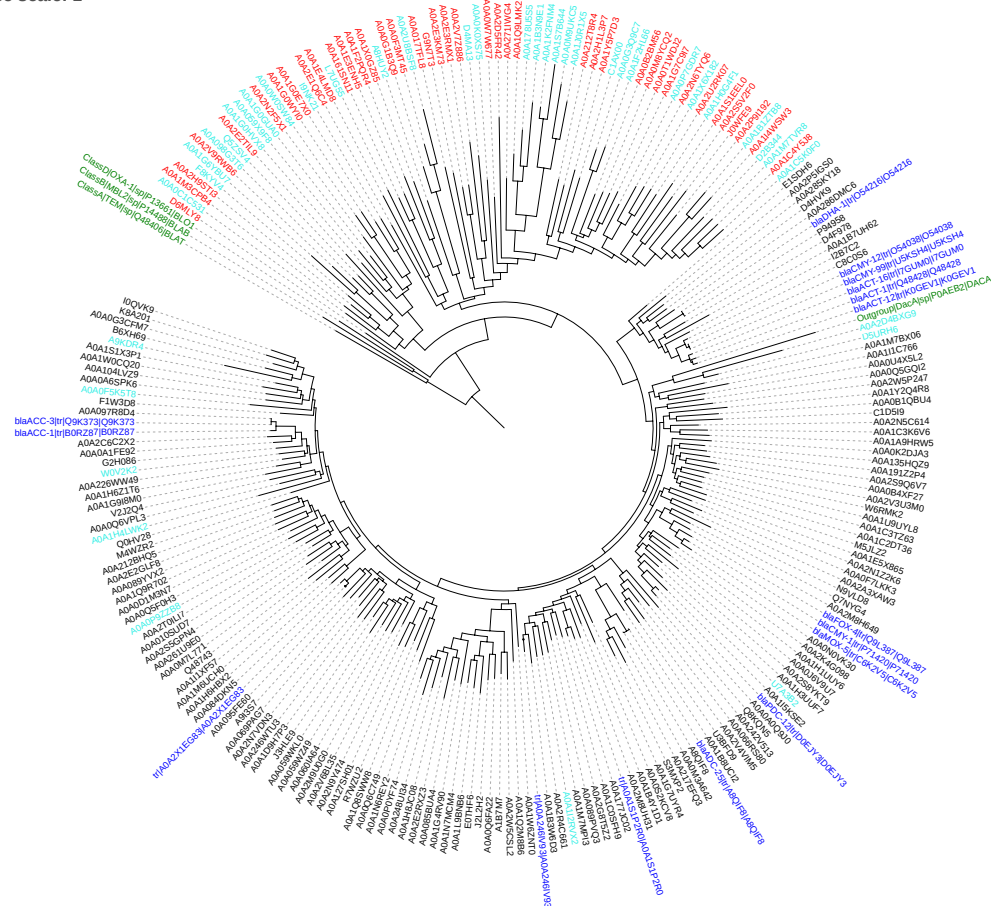**D**

Tree scale: 1

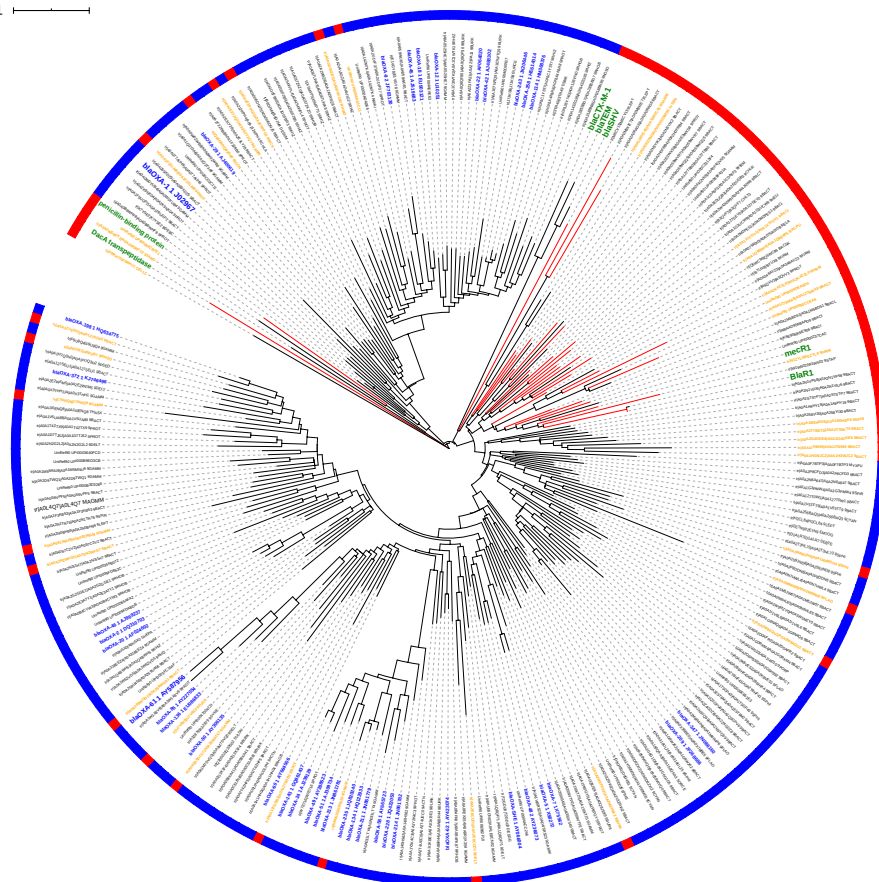

Supplement: FIG S1 [file msystems.01281-21-s0001.pdf]

A

316 TP\*

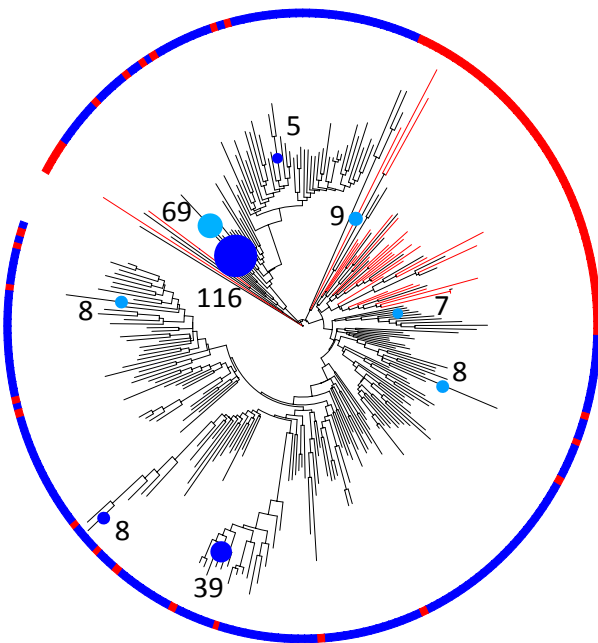

B

2 FN

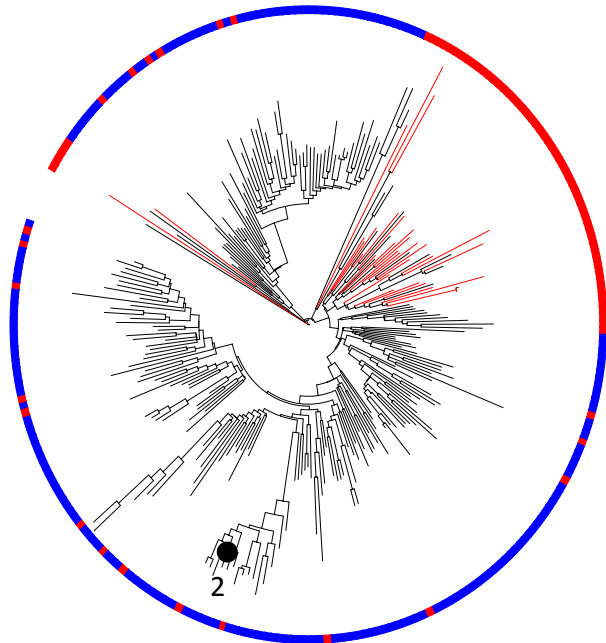

C

113 FP\*

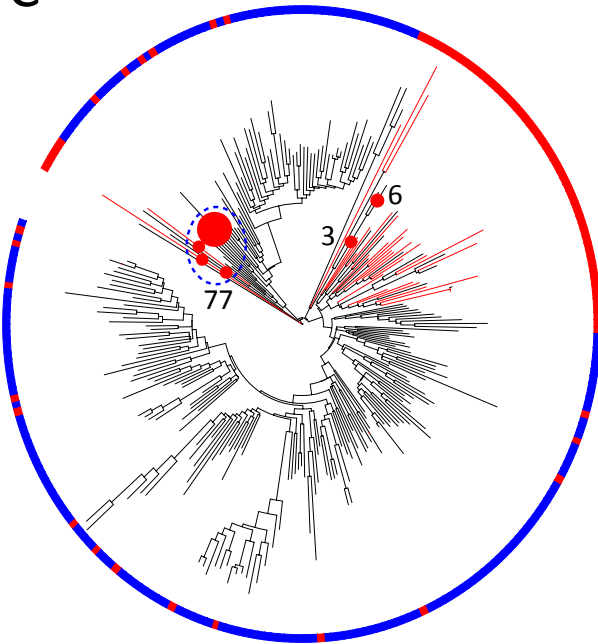

D

540 FP\*

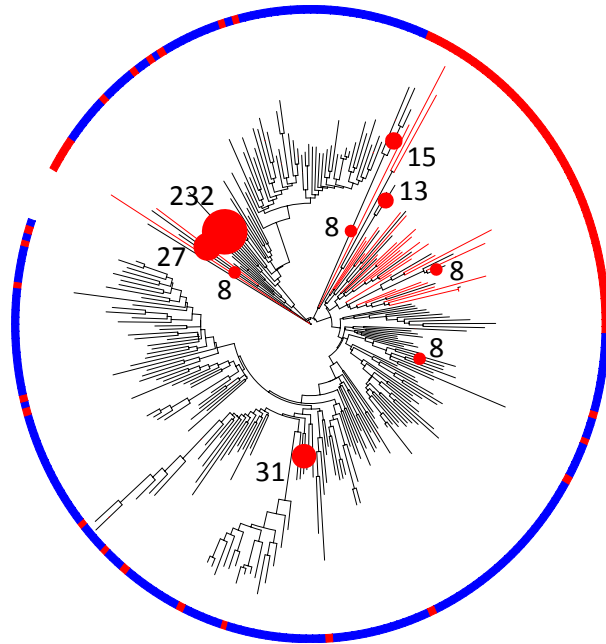

Tree Scale 1.0

Supplement: FIG S7 [file msystems.01281-21-s0007.pdf]

A

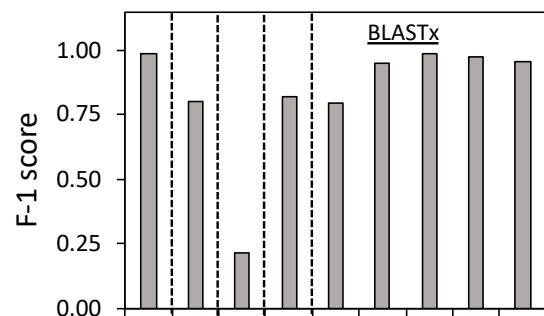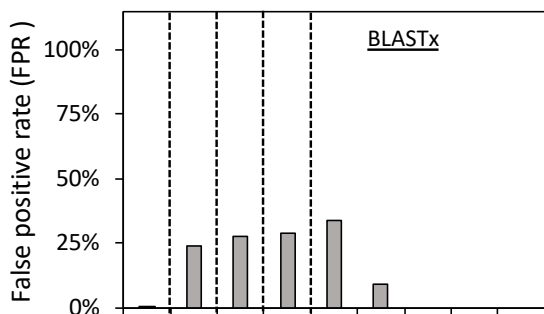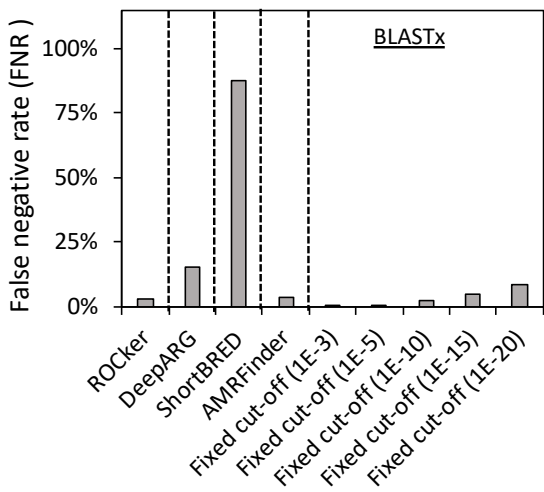

B

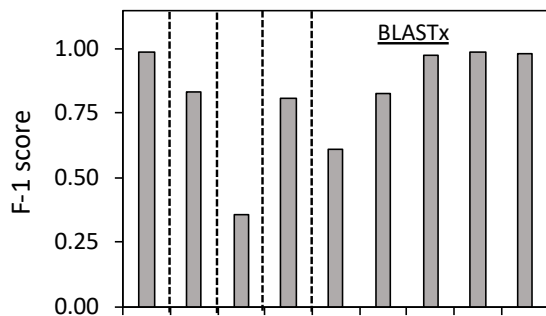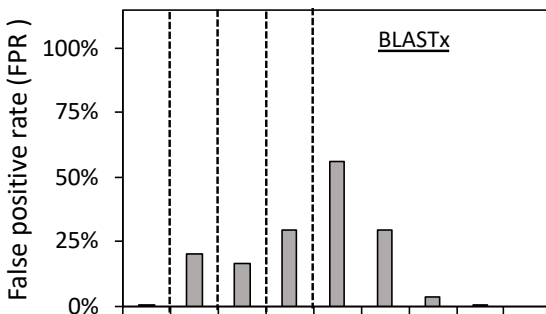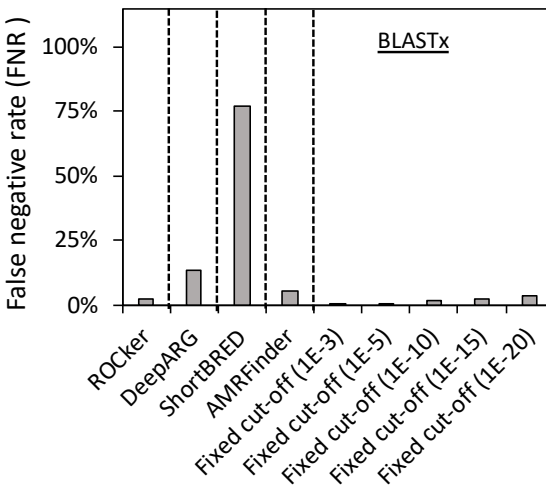

Supplement: FIG S8 [file msystems.01281-21-s0008.pdf]

**A**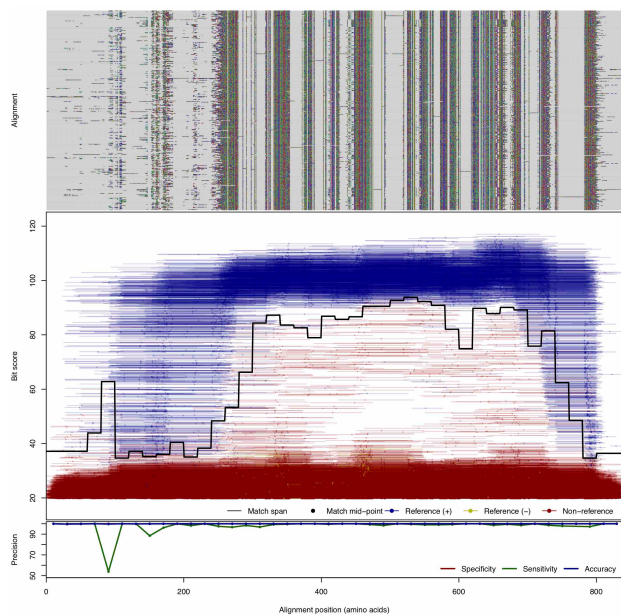**B**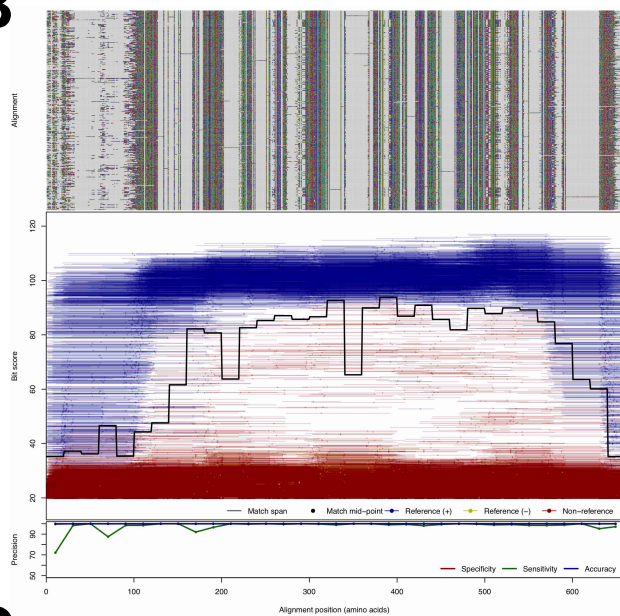**C**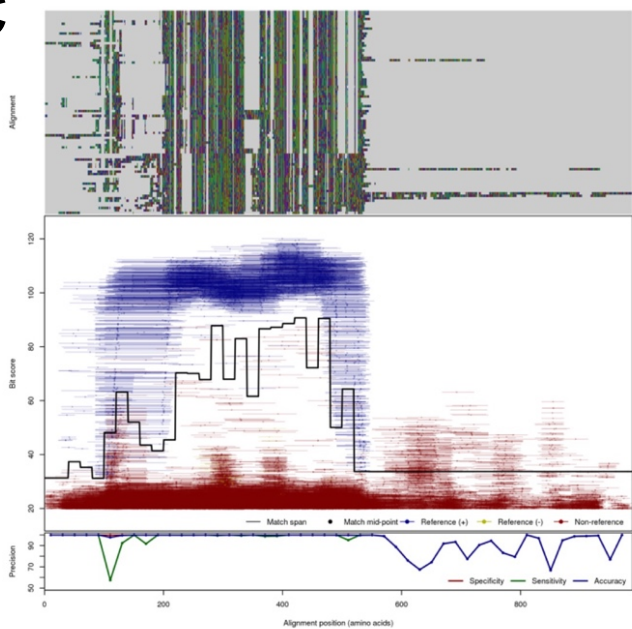**D**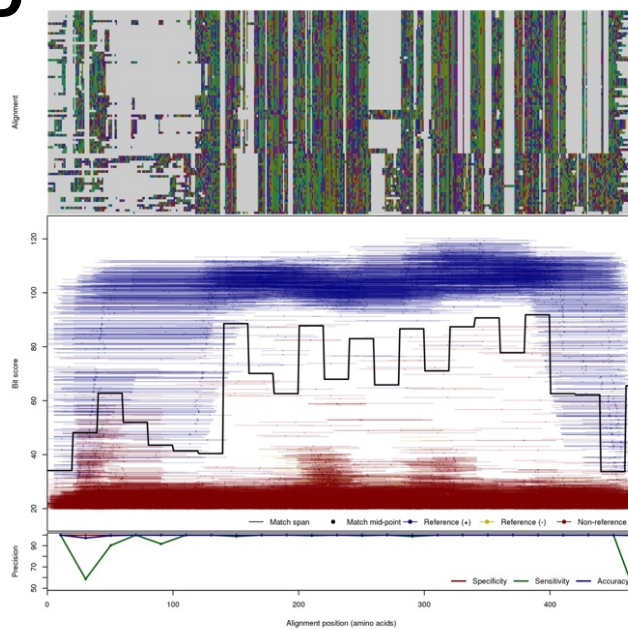**E**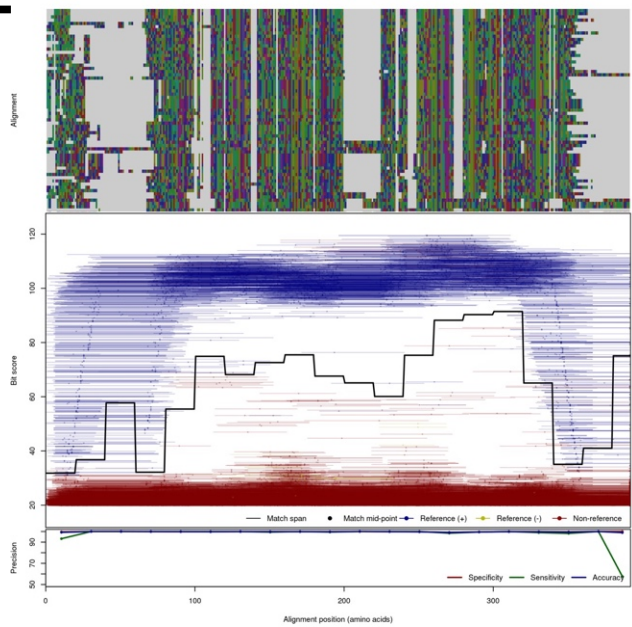**F**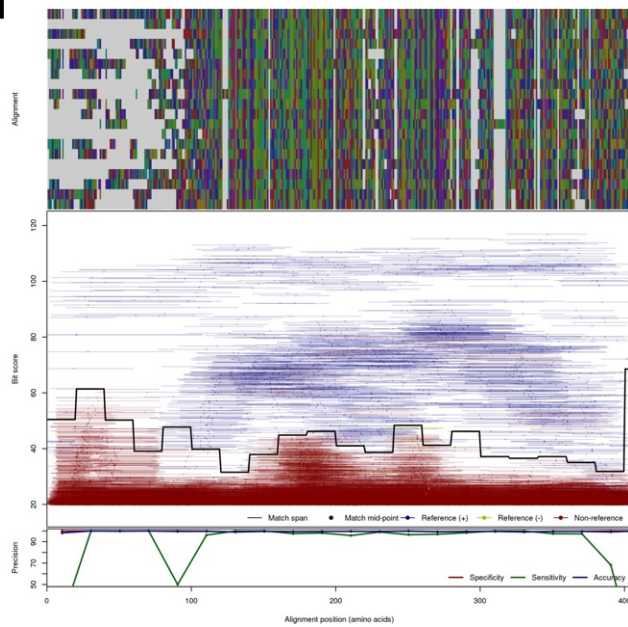

Supplement: FIG S3 [file msystems.01281-21-s0003.pdf]

**A**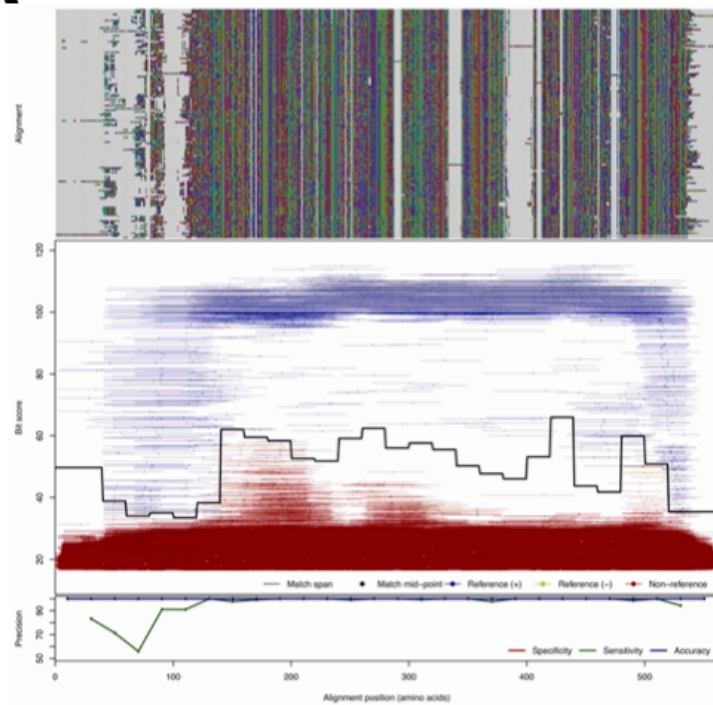**B**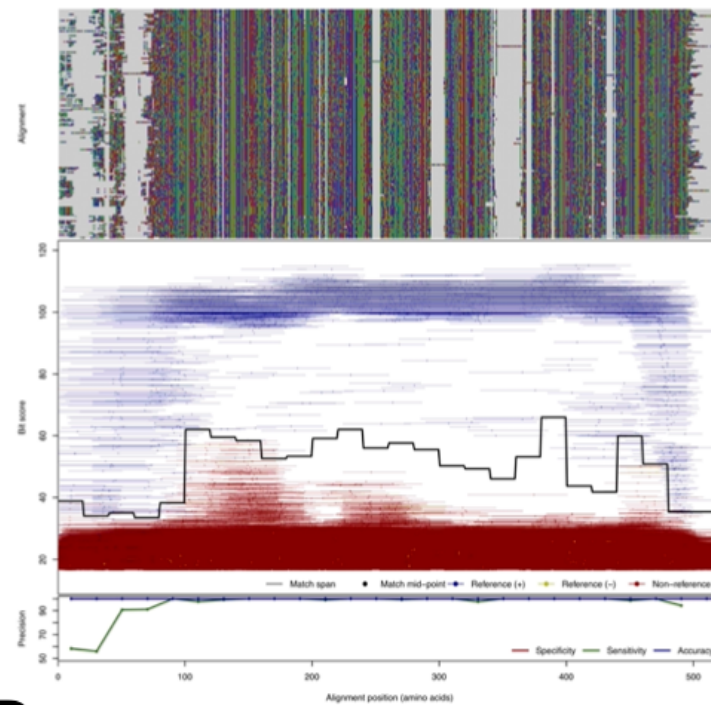**C**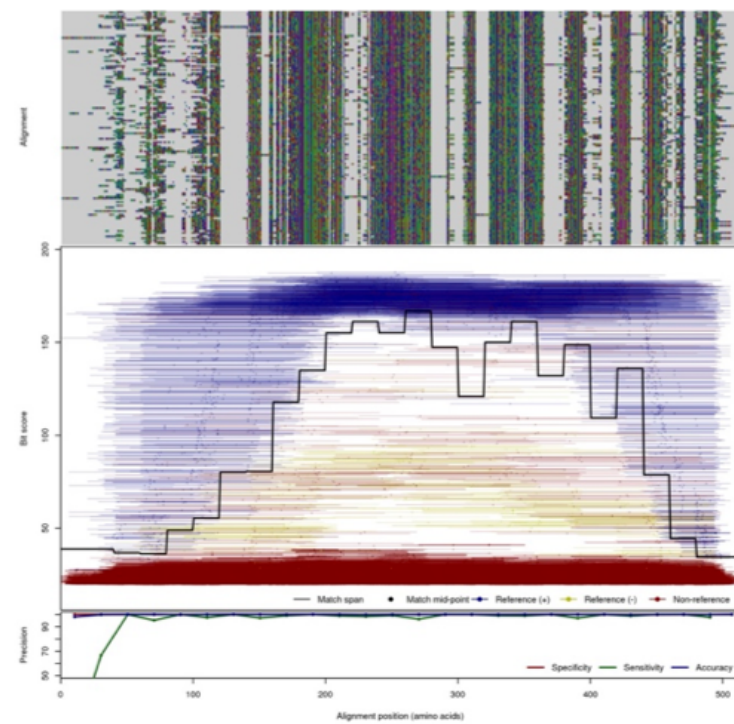**D**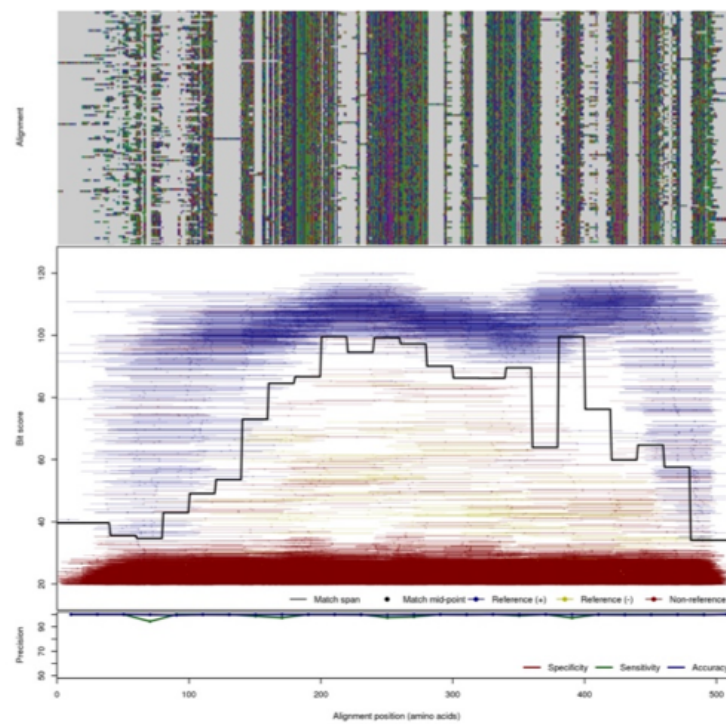

Supplement: FIG S4 [file msystems.01281-21-s0004.pdf]
