# Supplementary material for: ROCker Models for Reliable Detection and Typing of Short-Read Sequences Carrying β-Lactamase Genes
Source: mSystems. 2022 May 31;7(3):e01281-21. doi: 10.1128/msystems.01281-21 (PMC9238382; doi:10.1128/msystems.01281-21)
Supplement: TEXT S1 [file msystems.01281-21-s0010.docx]

**ROCker models for reliable detection and typing of short read sequences carrying β-lactamases**

Si-Yu Zhang^1,2*^, Brittany J. Suttner^1*^, Luis M. Rodriguez-R^1,3,4*^, Luis H. Orellana^5^, Roth E. Conrad^3^, Fang Liu^6^, Jessica L. Rowell^7,8^, Hattie E. Webb^7,8^, Amanda J. Williams-Newkirk^7^, Andrew Huang^7^, Konstantinos T. Konstantinidis^1,3^

1. School of Civil and Environmental Engineering, Georgia Institute of Technology, Atlanta, GA 30332, USA

2. School of Ecological and Environmental Sciences, East China Normal University, Shanghai, 200241, China

3. School of Biological Sciences, Georgia Institute of Technology, Atlanta, GA 30332, USA

4. Department of Microbiology and Digital Science Center (DiSC), University of Innsbruck, Innsbruck, Tyrol, Austria

5. Max-Planck-Institut für Marine Mikrobiologie, Bremen, Germany

6. Partnership for an Advanced Computing Environment (PACE), Georgia Institute of Technology, Atlanta, GA 30332, USA

7. Enteric Diseases Laboratory Branch, Centers for Disease Control and Prevention, Atlanta, GA, USA

8. Weems Design Studio, Suwanee, GA, USA

*These authors contributed equally to this work.

Corresponding author: Konstantinos T. Konstantinidis

E-mail: [kostas@ce.gatech.edu](mailto:kostas@ce.gatech.edu)

**MATERIALS AND METHODS**

**Determine the reference protein sequences for building the class A, B and C ROCker models**

**For class A BLs (Figure S1A),** the verified class A BL sequences were used to search against the UniRef90 database. Matches with >70% query length coverage by the alignments were de-replicated at 50% identity using cd-hit and one representative per resulting cluster was used, along with the verified class A BLs and non-targets proteins, to build the phylogenetic tree shown using FastTree version 2.1.7 (approximately-maximum-likelihood tree). FastTree was used here because of its computational efficiency in building large phylogenies, with little (and in some cases no) degradation in tree accuracy, as compared to RAxML. A more relaxed alignment length cut-off for finding putative class A BLs in UniRef90 was used (70%) compared to other BLs (90%) in order to capture as many class A BL sequences as possible given that their sequence length varied substantially between 266 - 375 amino acids. During model building, we noticed that some genomes of the positive references downloaded from UniProt database, which are used to simulate reads for the training data set creation step, carry more than one class A BL. These sequences caused the low accuracy of the resulting model due to not being tagged as positive references by ROCker. To deal with this issue, four additional class A BLs (Uniprot ID: C6YWK1, B0TZF6, A0A0B3VEE7 and A0EL75), which were carried by the same genomes carrying class A BL C6YW16, B0TXT1, A0A0B3VPP7 and Q7BEB3, were included in the positive reference set. Proteins from UniRef90 database that were missing at least one of the three conserved domains have the orange labels and were removed from the ROCker model building (except for those with red branches). Proteins highlighted in orange backgrounds were also removed from ROCker model building because they did not form a branch with either verified target or non-target proteins (thus, it was challenging to decide on the substrate specificity of such sequences). Red branches indicate proteins that were included in the negative reference set for ROCker model building because they formed a branch with verified non-target proteins; the remaining sequences (blue and black labels) were included in the positive set.

**For class B BLs (Figure S1B),** the verified MBL subtypes 1, 2, and 3 sequences were used to search against the UniProt90 database. Matches with >90% query length coverage by the alignments were de-replicated at 50% identity using cd-hit and one representative per resulting cluster was used, along with the verified MBLs and non-targets proteins (green labels), to build the phylogenetic tree shown using the maximum likelihood algorithm as implemented in RAxML version 7.7.2. Leaf labels highlighted with yellow background are functionally verified outgroups and were included in the negative reference set for ROCker model building along with proteins highlighted in orange. Red labels are missing at least one conserved domain and were excluded from model building (because it was challenging to decide on the substrate specificity of such sequences), except for the ones highlighted in yellow, which were included in the negative reference set for ROCker model building along with the functionally verified outgroups (green labels). Only sequences in pink and blue branches were included in the positive reference set in ROCker model building for the total MBL model and for the separate models by subtype (see below).

**For class C BLs (Figure S1C),** the verified class C BL sequences were used to search against the UniRef90 database. Matches with >90% query length coverage by the alignments were de-replicated at 60% identity using cd-hit and one representative per resulting cluster were used, along with the verified class C BLs and non-targets proteins, to build the phylogenetic tree shown using approximately-maximum-likelihood algorithm as implemented in FastTree version 2.1.7 (approximately-maximum-likelihood tree). FastTree was used here because of its computational efficiency in building large phylogenies, with little (and in some cases no) degradation in tree accuracy, as compared to RAxML. The matching (putative) class C BL sequences were de-replicated at a higher amino acid identity (60%) compared to other BLs (50%) in order to include as many class C BLs as possible given that some regions of class C BL sequences show relative low sequence identity within the class, i.e. around 30% amino acid identity. During model building, we noticed that some genomes of the positive and negative references downloaded from UniProt database, which are used to simulate reads for the training data set creation step, carry more than one class C BL and multiple proteins that closely related to the class C BLs, sharing around 30% identity to true class C BLs. These sequences caused the low accuracy of the resulting model due to not being tagged appropriately by ROCker (e.g., a class C BL was treated as a negative sequence during creating data sets for training purposes). To deal with this issue, three additional class C BLs (Uniprot ID: A0A0A1FAN2, A0A191YQC7 and P00811), which were carried by the same genomes carrying protein A0A0A1FE92 (class C BL), A0A191Z2P4 (class C BL) and P0AEB2 (DacA), were included in the positive reference set. The opposite also occurred a few times. That is, protein sequences forming a branch with verified non-target proteins, if their corresponding genomes carried proteins closely related to class C BLs, these negative sequences were marked in cyan labels and removed from the ROCker model building because the class BLs co-occurring in the genome caused the lower the accuracy of ROCker (or required manual work to remove or tag appropriately these sequences). The remaining sequences were marked in red labels and were included in the negative reference set for ROCker model building. Proteins forming a deep branch within the clade of verified class C BLs were also removed from model building if they were missing at least one of the three conserved domains (T[LI]F^60^[ED][LIV]G**S[VIL]SK**, RxY^150^xN and K^315^TG) (cyan labels), as it was challenging to decide on the substrate specificity of such sequences (conservative approach). The blue and black labels in Figure S1C indicate proteins that included in the positive set.
