# Supplementary material for: ROCker Models for Reliable Detection and Typing of Short-Read Sequences Carrying β-Lactamase Genes
Source: mSystems. 2022 May 31;7(3):e01281-21. doi: 10.1128/msystems.01281-21 (PMC9238382; doi:10.1128/msystems.01281-21)
Supplement: FIG S5 [file msystems.01281-21-s0005.pdf]

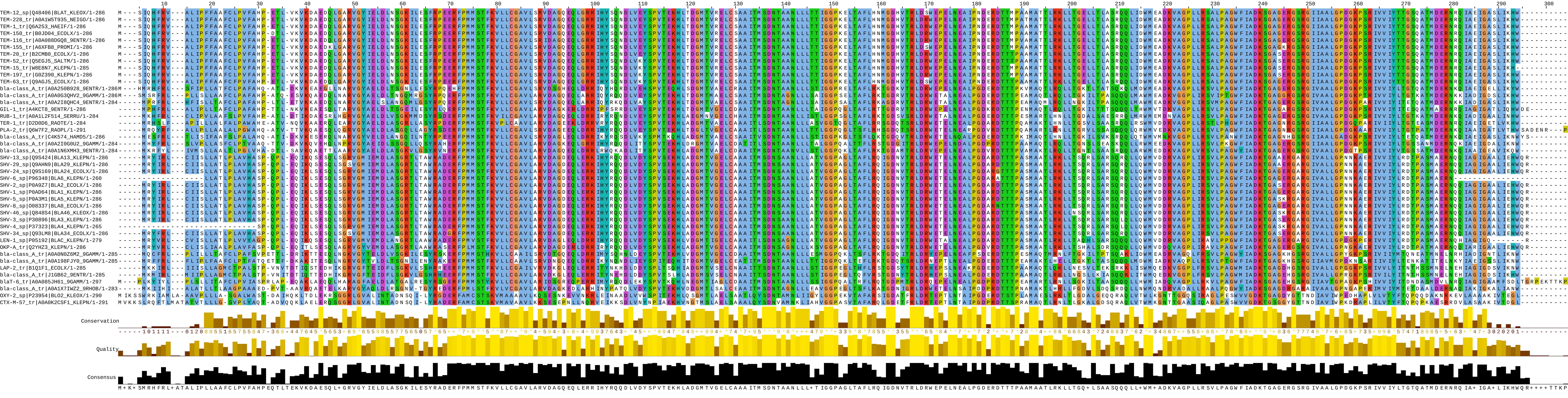
TEM-12_sp|Q48406|BLAT_KLEOX/1-286
TEM-228_tr|A0A1W5T935_NEIGO/1-286
TEM-1_tr|Q6A253_HAEIF/1-286
TEM-150_tr|B0JD04_ECOLX/1-286
TEM-116_tr|A0A088DGQ8_9ENTR/1-286
TEM-155_tr|A6XFB8_PROMI/1-286
TEM-20_tr|B2CMB0_ECOLX/1-286
TEM-52_tr|Q5EGJ5_SALTM/1-286
TEM-15_tr|W8E8N7_KLEPN/1-285
TEM-197_tr|G0Z390_KLEPN/1-286
TEM-63_tr|Q9AGJ5_ECOLX/1-286
bla-class_A_tr|A0A250B928_9ENTR/1-286
bla-class_A_tr|A0A0G3QHV2_9GAMM/1-286
bla-class_A_tr|A0A2I8QHC4_9ENTR/1-284
GIL-1_tr|A4KCT8_9ENTR/1-286
RUB-1_tr|A0A1L2F514_SERRU/1-284
TER-1_tr|D2D0D6_RAOTE/1-284
PLA-2_tr|Q6W7F2_RAOPL/1-291
bla-class_A_tr|C4K574_HAMD5/1-286
bla-class_A_tr|A0A2I0G0U2_9GAMM/1-284
bla-class_A_tr|A0A1N6XMH3_9ENTR/1-284
SHV-13_sp|Q9S424|BLA13_KLEPN/1-286
SHV-29_sp|Q9AHN9|BLA29_KLEPN/1-286
SHV-24_sp|Q9S169|BLA24_ECOLX/1-286
SHV-6_sp|P96348|BLA6_KLEPN/1-260
SHV-2_sp|P0A9Z7|BLA2_ECOLX/1-286
SHV-1_sp|P0AD64|BLA1_KLEPN/1-286
SHV-5_sp|P0A3M1|BLA5_KLEPN/1-286
SHV-8_sp|O08337|BLA8_ECOLX/1-286
SHV-46_sp|Q848S4|BLA46_KLEOX/1-286
SHV-3_sp|P30896|BLA3_KLEPN/1-286
SHV-4_sp|P37323|BLA4_KLEPN/1-265
SHV-34_sp|Q93LM8|BLA34_ECOLX/1-286
LEN-1_sp|P05192|BLAC_KLEPN/1-279
OKP-A_tr|Q2YHZ3_KLEPN/1-286
bla-class_A_tr|A0A0N0Z6M2_9GAMM/1-285
bla-class_A_tr|A0A198FJY0_9GAMM/1-285
LAP-2_tr|B1Q1F1_ECOLX/1-285
bla-class_A_tr|J1GB62_9ENTR/1-285
blaT-6_tr|A0A085JH61_9GAMM/1-297
bla-class_A_tr|A0A1X7IWZ2_9RHOB/1-283
OXY-2_sp|P23954|BLO2_KLEOX/1-290
CTX-M-57_tr|A0A0K2CSF1_KLEPN/1-291
Conservation
Quality
Consensus
